# Dynamic mechanical response and failure characteristics of coal and rock under saltwater immersion conditions

**DOI:** 10.1038/s41598-024-62596-w

**Published:** 2024-05-24

**Authors:** Xiaoyuan Sun, Kai Liu, Tingxu Jin, Kai Wang, Shurong Lin, Jiewen Pang, Jianlin Xie

**Affiliations:** 1 College of Safety and Emergency Management Engineering, Taiyuan University of Science and Technology, Taiyuan, 030024 Shanxi People’s Republic of China; 2 Intelligent Monitoring and Control of Coal Mine Dust Key Laboratory of Shanxi Province, Taiyuan University of Science and Technology, Taiyuan, 030024 Shanxi People’s Republic of China; 3 School of Emergency Management and Safety Engineering, China University of Mining and Technology (Beijing), Beijing, 100083 People’s Republic of China; 4 School of Civil and Mechanical Engineering, Curtin University, Perth, WA 6845 Australia

**Keywords:** Saltwater, Water-bearing coal and rock mass, Dynamic mechanical response, Failure characteristic, Fractal dimension, Civil engineering, Coal

## Abstract

The stability of coal and rock masses in water-rich mines is affected by both mine water erosion and dynamic disturbances. Thus, it is necessary to study the dynamic mechanical response and failure characteristics of coal and rock under the combination of saltwater and a high strain rate. To this end, a split Hopkinson pressure bar device was employed to investigate the effects of impact velocity, water content, and immersion liquid on the dynamic mechanical behaviours of coal and rock. The results revealed that the weakening effect of saltwater on the dynamic mechanical properties of coal and rock is much greater than that of distilled water. With increasing moisture content, the dynamic compressive strength of the coal specimens decreases monotonically, while that of the rock shows a trend of first increasing and then decreasing. The failure process and destruction of coal and rock are comprehensively affected by both the external impact load and the physical and mechanical properties of the material. The degree of damage of the coal and rock specimens increases with increasing impact velocity and water content. Moreover, the influence of various factors on the impact fracture mechanism of coal and rock under saltwater immersion conditions was revealed. These findings are highly important for the design and maintenance of underground coal and rock building structures.

## Introduction

In typical underground engineering operations, coal mines are frequently affected by water during mining. This water includes atmospheric precipitation and surface water that enters underground through various water-conducting channels, as well as groundwater represented by aquifer water, fault fissure water, and goaf water^1^. On the one hand, the presence of water can weaken the strength of coal pillars^2^, induce roof accidents^3^, and even directly flood mining spaces and cause water inrush accidents^4^. On the other hand, technologies such as coal seam water injection^5,6^, water jetting^7,8^, and goaf grouting^9,10^ have been widely used for compulsive roof cave-in^11,12^, stress balance ahead of the mining face^13^, increased coal seam permeability^14^, suppressed rockburst^15^ and coal and gas outbursts^16^, and reducing mine fires^17^ and dust disasters^18^. Furthermore, to alleviate the prominent contradiction between mining and water resource protection in environmentally fragile mining areas in western China, some scholars have proposed using coal mines as underground water storage spaces^19–21^. In this case, a comprehensive understanding of the mechanical response and failure characteristics of coal and rock under water-bearing conditions is highly important for ensuring safe production and disaster warning in coal mines.

In recent decades, many mechanical tests have been conducted on coal and rock under different water content conditions^22^, and fruitful results have been achieved. Destructive loading methods include uniaxial compression^23,24^, triaxial compression^25,26^, Brazilian splitting^27,28^, four-point bending^29^, and notched semicircular bending (NSCB)^30,31^ of water-bearing coal and rock. In general, as the moisture content increases, the strength, stiffness, elastic modulus and other physical parameters of coal and rock mostly tend to decrease, while the crack propagation speed and specimen failure morphology become more complex. The reasons for this can be summarized as follows^32,33^: (1) decreasing surface free energy, (2) reducing internal friction, (3) increasing pore pressure and (4) deteriorating physical and chemical properties.

Notably, the above research results were all obtained under static or quasistatic conditions. In fact, due to strong disturbances such as rapid excavation and coal blasting in coal mines^34^, analysing the dynamic damage and destruction of water-bearing coal and rock masses under impact loads is also highly important. The mechanical properties and failure characteristics of coal and rock masses under static and dynamic loads are completely different^35^. Man et al.^36^ conducted static compression and split Hopkinson pressure bar (SHPB) dynamic loading experiments on naturally air-dried and water-saturated granite, respectively, and found that the compressive strength of naturally air-dried granite was greater than that of water-saturated specimens under static load conditions but showed a completely opposite pattern under dynamic loading conditions. Gu et al.^37–39^ conducted impact experiments on coal specimens with different water contents and porosities, and elucidated the influential mechanism of the above factors on the crack propagation and dynamic compressive strength of soft coal. Zhang et al.^40^ found that the presence of water can slow the transmission of stress waves and the deformation of specimens, thereby enhancing the dynamic tensile strength of mortar. Li et al.^22^ conducted SHPB impact tests on sandstones subjected to different wet‒dry cycles and found that as the number of cycles increased, the porosity of the specimens increased, while the dynamic strength and total energy consumption decreased. Wang et al.^41^ utilized a homemade experimental device to test the strength and failure type of coal specimens under the coupled conditions of static axial preloading, impact loading, and water pressure, and found that water could increase the dynamic strength of the specimens and reduce the failure strain of the specimens.

Previous studies have investigated the effects of water and dynamic load disturbances on the mechanical behaviour and failure characteristics of coal and rock. However, it should be noted that the water used in the above experiments was mostly distilled water or untreated tap water. Mine water is actually multiphase composite water^42,43^, in which the dominant components of inorganic minerals are Na^+^, K^+^, Ca^2+^, Cl^−^, CO_3_^2−^, and SO_4_^2−^^44,45^. Studies have shown that the influence of NaCl solution on the mechanical properties of coal and the degree of specimen breakage is greater than that of distilled water under dynamic impact loading^46,47^. There have been relatively few studies on the characterization of the high strain rate of coal and rock under the coupled condition of dynamic load and water content, and the effects of dynamic load and NaCl solution on the mechanical properties and failure characteristics of coal and rock. Furthermore, more information on the real-time deformation and fracture evolution of coal and rock specimens containing NaCl solution under impact dynamic loading conditions needs to be obtained.

In this paper, first, the pretreatment of coal and rock specimens by immersion in distilled water and NaCl saline solution was carried out, and a SHPB impact experimental platform and high-speed data processing and image acquisition system were established. Then, based on the methods of stress‒strain signal processing, box dimension and mass dimension calculations, the dynamic mechanical response, energy dissipation and damage characteristics under different impact velocities, water contents and brine immersion conditions were determined. Finally, the influential mechanism of distilled water and saline water on coal and rock dynamics was characterized from a microscopic perspective by using scanning electron microscopy (SEM) and mathematical analysis. The research results will help to supplement and improve the dynamic load stability of coal or rock tunnel engineering under water immersion conditions and provide scientific support for the safe design and operation maintenance of related underground coal and rock building structures.

## Materials and methods

### Specimen preparation

**Figure 1:**
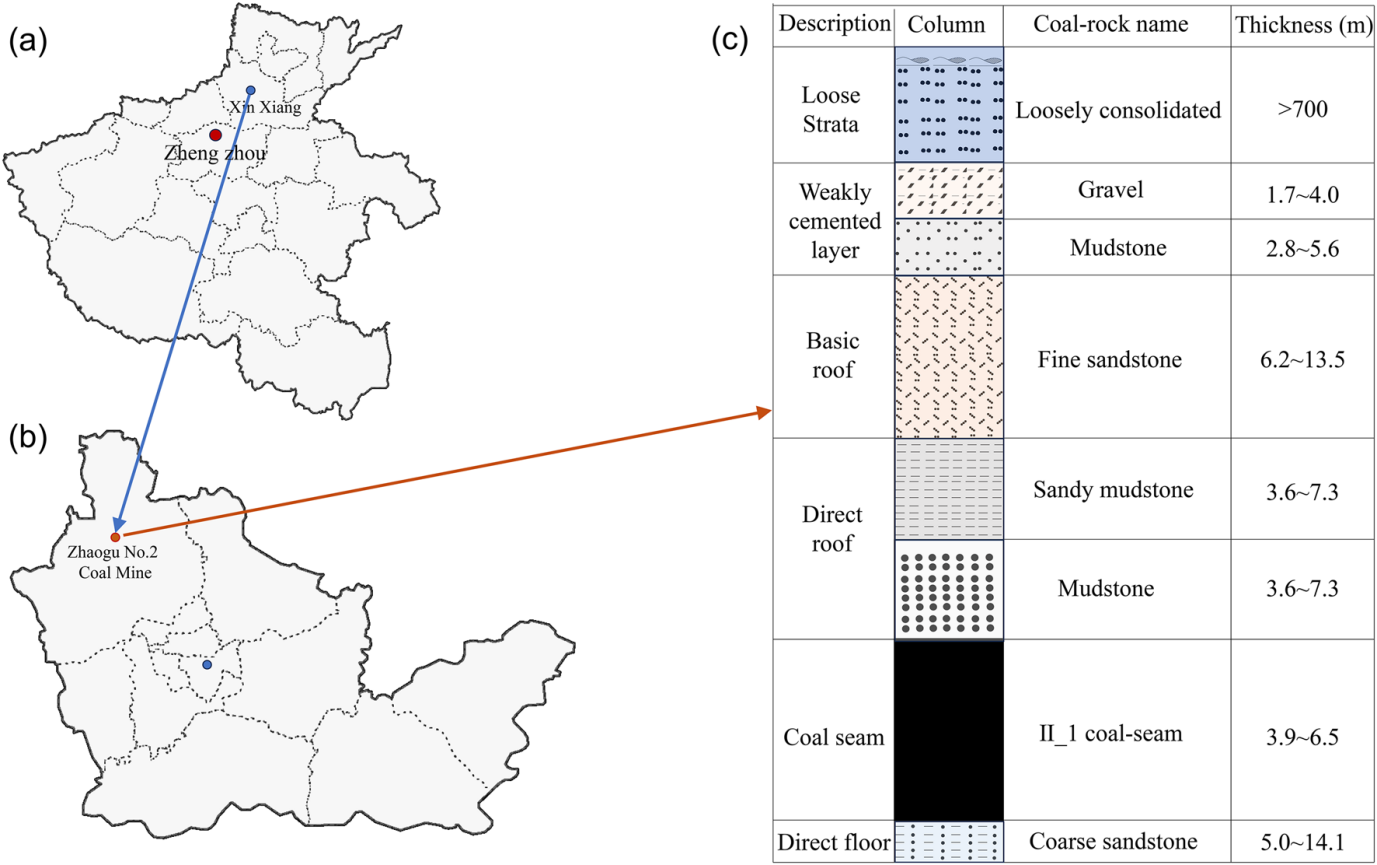
Schematic diagram of the coal and rock sampling. (**a**) Geographical location of Xinxiang city; (**b**) location of the Zhaogu No. 2 Coal Mine; and (**c**) comprehensive stratigraphic bar chart.

As illustrated in Figs. 1a,b, the coal and rock specimens were collected from a coal seam and basic roof of the 14,030 working face in the Zhaogu No. 2 Coal Mine of Xinxiang city, China. The comprehensive horizontal bar chart is shown in Fig. 1c. The 14,030 working face is characterized by deep burial, thick alluvium, weak cementation, thin bedrock and a thick coal seam^48^. Research has shown that the above mining conditions exhibit the following characteristics: (1) The alluvial layer contains multiple aquifers, and the mining area is located in an area where the Yellow River tributary flows, with a high groundwater level^49,50^. With increasing mining height and complete collapse, the height of the water-conducting fractured zone significantly increases^51^, further increasing the possibility of the alluvial layer of groundwater flowing along the mining fracture towards the working face and goaf and even posing a threat of sudden water and sand inrush to the working face^52^. (2) The mining pressure in the working face is strong, the dynamic load coefficient is extremely high, and the dynamic load impact effect occurs from time to time^53^. In addition, due to the widespread external disturbances during mining, exploring the mechanical response and failure characteristics of coal and rock masses under the coupled effect of mine water and impact dynamic loads has strong practical significance and pertinence.

**Figure 2:**
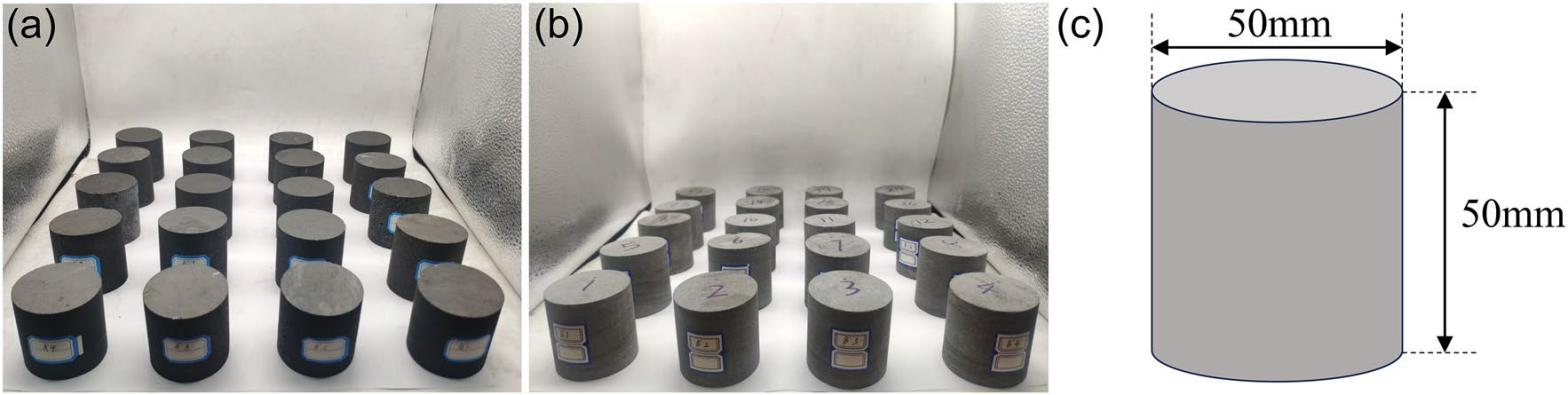
Illustration of the coal and rock specimens used in the experiment. (**a**) RCSs; (**b**) RRSs; and (**c**) illustration of specimen size.

According to the standards recommended by the International Society for Rock Mechanics and Rock Engineering (ISRM)^54^, cylindrical specimens were drilled from coal seams and basic roof strata collected at the same location, with a length of 50 mm and a length: diameter ratio of 1:1. Subsequently, polishing was carried out to maintain the surface roughness of the specimens within 0.02 mm, and the difference between the normals of the end faces and the long axis was reduced to less than 0.001 rad. In this experiment, a total of 20 raw coal specimens (RCSs) and 15 dark grey sandstone specimens (raw rock specimens, RRSs) were prepared, as shown in Fig. 2.

### Experimental system

**Figure 3:**
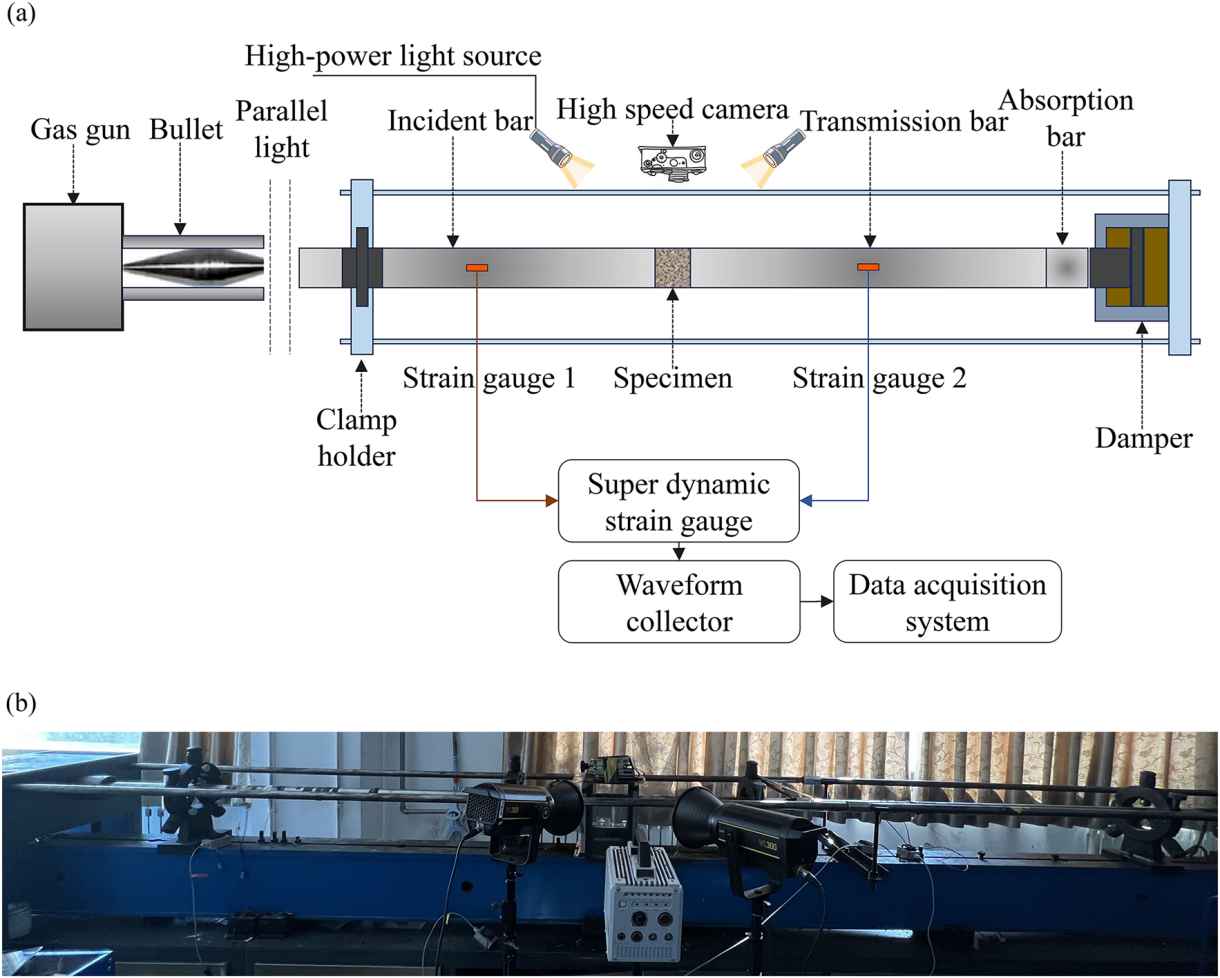
Diagram of the experimental system. (**a**) Schematic diagram and (**b**) physical diagram.

The main equipment used in this experiment was the SHPB impact loading system developed by the China University of Mining and Technology (Beijing). As shown in Fig. 3a, the system consists of three main parts, i.e., a stress wave generator, a bar system, and a data processing and video acquisition unit. During an experiment, the stress wave generator starts to make a bullet impact the incident bar at different speeds, thus generating a certain shape of the loading stress wave. The stress wave acts on the coal or rock specimen through the bar system. The bar system consists of an incident bar, a transmission bar, and an energy absorption bar, with the specific parameters shown in Table 1. To collect the transient signals generated by stress waves in the bar system, two strain gauges were affixed to the middle of the incident bar and projection bar, respectively. The signals were transmitted to the ultradynamic strain gauge, waveform acquisition unit, and data processing system for analysis, thus obtaining various parameters of the specimen.

**Table 1:** Bar system parameter table.

Device name		Density (kg/m^3^)	Elastic modulus (Gpa)	Diameter (mm)	Length (mm)
Bar system	Incident bar	7740	206	50	3000
	Transmission bar				2500
	Energy absorption bar				1000

To capture the damage images of coal and rock during SHPB impacts, the data processing and video acquisition unit shown in Fig. 3b also included an ultrahigh-speed camera and a high-brightness flood light, which is convenient for effectively revealing the details of crack evolution during coal and rock damage^34^. The specific equipment parameters are shown in Table 2. In addition, the perimeter of the coal and rock specimens was surrounded by a high-transmittance acrylic plate, which prevents splashing debris from damaging the camera while also facilitating the collection and screening of damaged specimens. During the experiment, the optical axis of the camera was controlled to be consistent with the normal of the specimen surface, and the angle of the flood light was adjusted multiple times to minimize the influence of the acrylic plate spot and improve the experimental accuracy.

**Table 2:** Ultrahigh-speed camera and high-light filling light parameter table.

Device name	Name of parameter	Correlation parameter values
Ultrahigh-speed camera	Model	FASTCAM SA5
	Experimental resolution	320 × 192
	Image frame frequency	100,000 FPS
	Shooting interval	10 μs
High-brightness flood light	Model	VL 300
	Colour temperature	5600 ± 200 K
	Illuminance (assembly with a reflector)	77,000 lx/m

**Figure 4:**
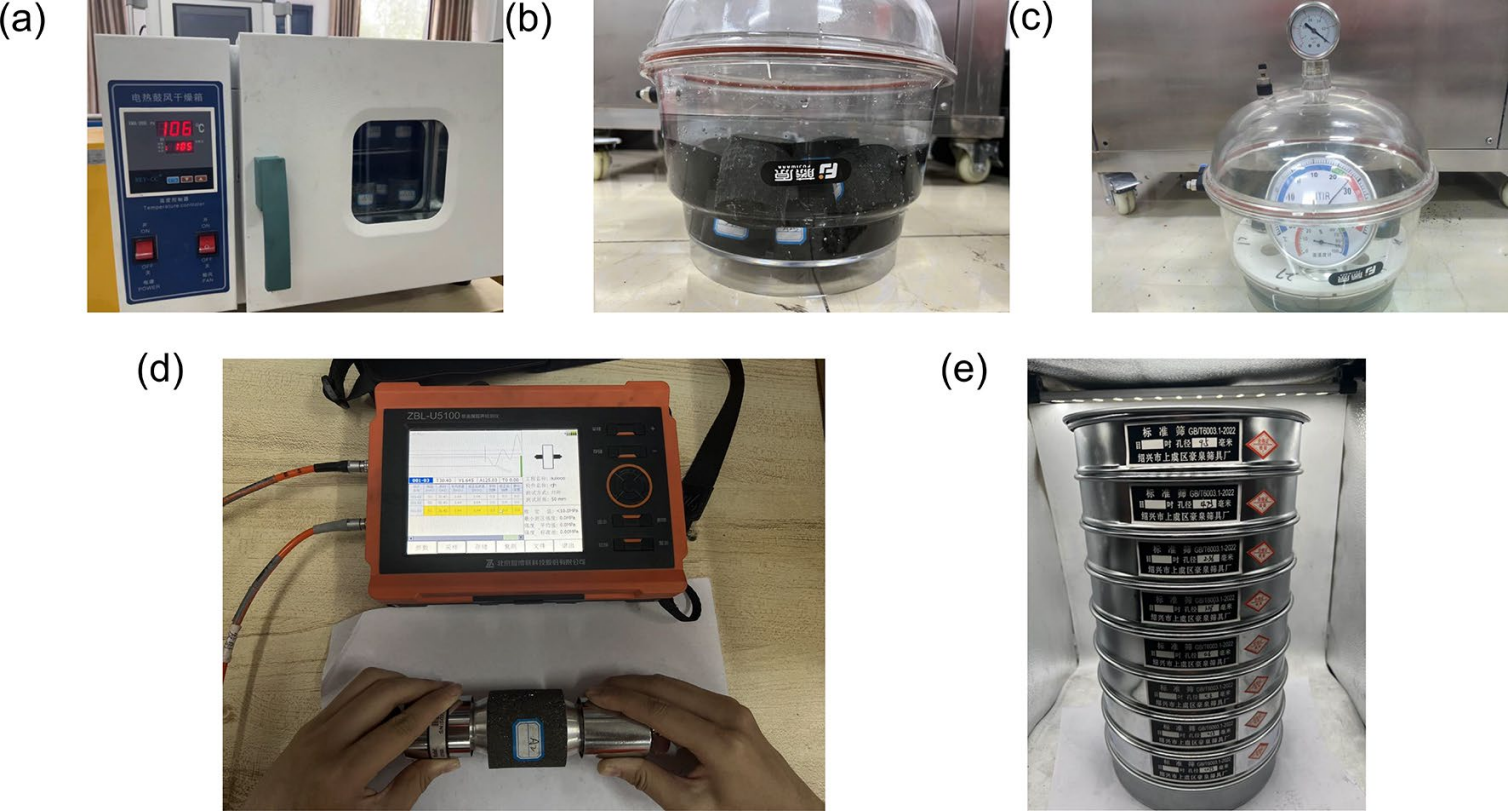
Auxiliary experimental equipment. (**a**) Drying oven; (**b**) liquid container (saturated state); (**c**) liquid container (naturally hydrated state); (**d**) ultrasonic detector; and (**e**) specimen sieve.

Since the purpose of the experiment was to investigate the differences in the mechanical properties and failure characteristics of coal and rock under different distilled water and saltwater conditions, in addition to the main equipment mentioned above, a drying oven, a water container, an ultrasonic detector, and a specimen sieve were also used, as shown in Fig. 4. Figure 4a shows the electric blast drying oven, which is designed to dry coal and rock specimens while also controlling the moisture content of the specimens. The water containers, as shown in Fig. 4b,c, correspond to the saturated and naturally hydrated states, respectively. For a saturated state, it was necessary to soak the coal and rock mass. The liquid level was 1–2 cm above the top surface of the specimen. In contrast, for the naturally hydrated state, the coal and rock specimens must be placed on a perforated cover plate, and the bottom of a specimen should not directly contact the water surface. The above containers can be used not only to hold water but also to store NaCl solutions. Figure 4d shows the ZBL–U5100 nonmetallic ultrasonic detector produced by Beijing Zhibolian Science and Technology Co., Ltd., consisting of a transmitting end and a receiving end, which was used to determine the ultrasonic longitudinal wave velocity (C_P_) of the coal and rock specimens. The test was conducted in a ‘face-to-face’ manner^55^. Vaseline was applied to the ends of the instrument and specimen to ensure sufficient coupling while maintaining consistent pressure on the sensor each time, and measurements were taken multiple times to reduce experimental errors. Figure 4e shows the specimen sieve used in this experiment, which was used to divide the material into 8 different pore sizes according to relevant standards^56^, namely, 9.50 mm, 4.75 mm, 2.36 mm, 1.18 mm, 0.60 mm, 0.30 mm, 0.15 mm, and 0.075 mm.

### The process and principle of water absorption design for specimens

#### Process of water absorption design

**Figure 5:**
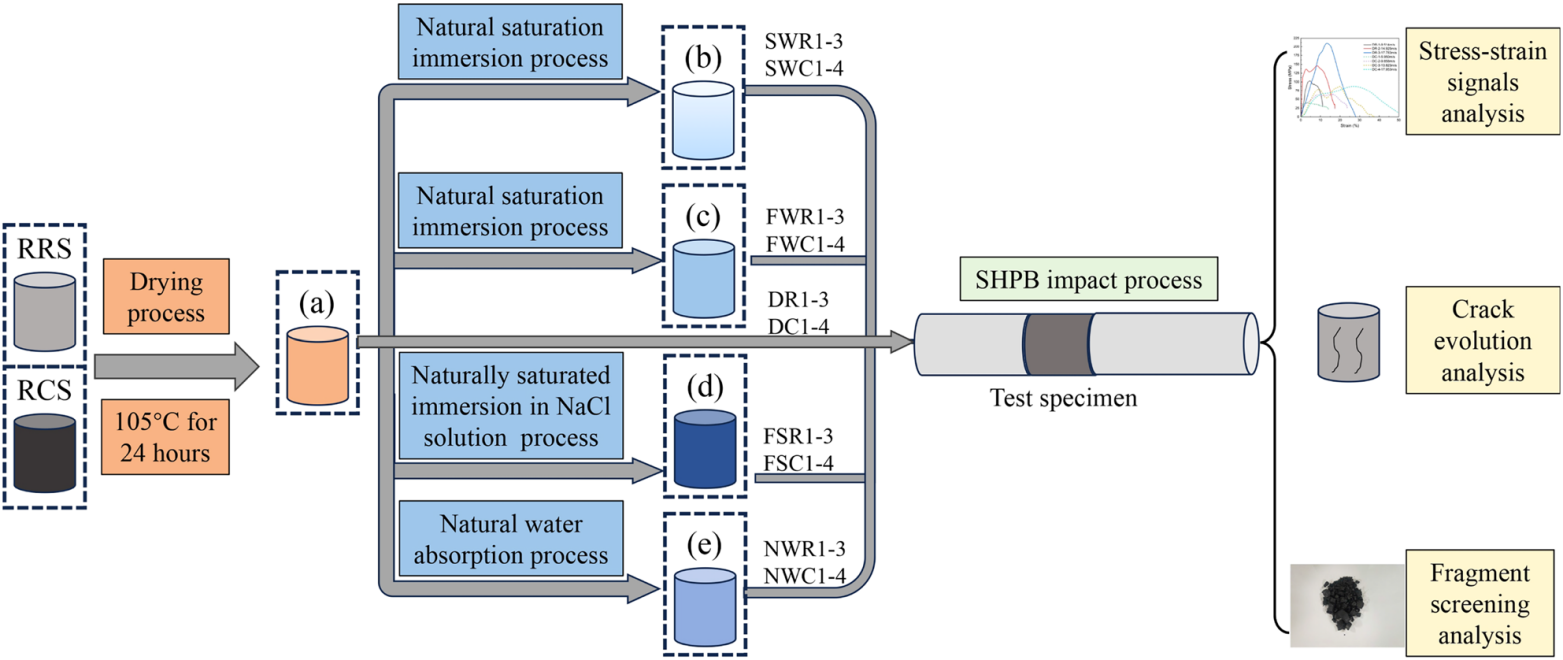
Experimental flow chart. (**a**) Drying status; (**b**) 50% moisture content status; (**c**) saturated water content status; (**d**) saturated NaCl solution status; and (**e**) natural moisture status.

Before the SHPB impact experiment, the specimens were pretreated, as shown in Fig. 5. Referring to the experimental process, all the specimens were dried in a drying oven at 105 °C for a minimum of 24 h^57^. Subsequently, the specimens were removed every hour and weighed using a high-precision balance until the mass difference between the two measurements was ≤ 0.01 g, indicating that the specimens met the drying requirements. Finally, the specimens were classified into five groups from I to V. Each group contained four RCSs and three RRSs. Group I specimens were not subjected to any secondary processing. The specimens in Groups II–IV were immersed in the water container shown in Fig. 2b for a minimum of 24 h and then weighed again until the difference between the two mass measurements was ≤ 0.01 g. The difference was that Group II specimens were redried in an oven after reaching a saturated state until the moisture content reached 50%. The liquid used to soak Group IV specimens was not water but rather a 0.1 mol/L NaCl solution^58–60^.Similarly, the Group V specimens were naturally absorbed in a container with water at the bottom and a perforated partition in the centre, as shown in Fig. 2c, for a minimum of 48 h and then weighed again until the difference between the two mass measurements was ≤ 0.01 g. Before and after each operation, the ultrasonic detector shown in Fig. 2d was used to measure the longitudinal wave velocity of the coal and rock specimens to reflect the influence of the water or brine status on the physio-mechanical properties of the solid medium.

Next, SHPB dynamic load failure experiments were conducted on the pretreated specimens. The coal and rock specimens were impacted at four and three different levels of velocity, respectively. During the impact process, stress‒strain signals and high-speed photographic videos were simultaneously recorded to reflect the dynamic response and crack evolution characteristics of the specimens. After impact, the coal and rock fragments were collected and sieved to conduct in-depth quantitative analysis of the fragmentation degree and to characterize the fragmentation degree at the microscopic level.

#### Principles of water absorption design

According to the relevant theories of hydrogeology, the forms of water in coal and rock voids usually include bound water, gravity water, and capillary water. Bound water is difficult to release before the temperature significantly increases, while gravity water and capillary water can evaporate at temperatures slightly higher than the evaporation temperature^61^. Assuming that the mass of a specimen after drying is $m_{d}$ and the mass of the specimen saturated with water is $m_{s}$, the expression $\omega_{t}$ for the saturation of the specimen after a certain water-containing treatment is:

1 $$ \omega_{t} = \frac{{m_{t} - m_{d} }}{{m_{s} - m_{d} }} \times 100\% $$

where $m_{t}$ is the mass of the specimen after some treatment. The saturation value is presented as a percentage. The 50% saturated specimen shown in Fig. 5b was prepared by first saturating the specimen with water and then dehydrating it at a constant temperature. Specifically, the first step was to dry the specimen and weigh the mass. Then, the specimen was saturated with water by immersion and weighed. The above values were substituted into Eq. (1), and the specimen mass when the left side was 50% was calculated. The saturated specimen was placed into a drying oven at 105 °C for drying, and its mass was measured using a high-sensitivity electronic scale every 20 min. When the specimen mass reached $m_{t}$, a semisaturated and water-containing specimen was obtained.

In addition, the water content is also commonly used in laboratory studies to reflect the relevant characteristics of coal and rock specimens^27^, and its expression is shown in Eq. (2):

2 $$ \omega_{{\text{W}}} = \frac{{m_{w} - m_{d} }}{{m_{d} }} \times 100\% $$

where $\omega_{w}$ is the water content of the coal or rock specimen and $m_{w}$ and $m_{d}$ are the masses of the specimen before and after drying treatment, respectively.

### Experimental principles

#### Principles of the SHPB test

As shown in Fig. 3a, the specimen is clamped between the incident bar and the transmission bar. During the experiment, the stress wave generator is released to cause the bullet to impact the incident bar at high speed, generating a stress pulse wave that propagates along the incident bar to the specimen. Due to the significant difference in wave impedance between the specimen and the bar, stress pulses reflect and transmit on both the left and right end faces of the specimen. After multiple transmissions and reflections, the stress values from both ends of the specimen are basically consistent. By collecting signals of incident $\varepsilon_{i} (t)$, reflected $\varepsilon_{r} (t)$, and transmitted $\varepsilon_{t} (t)$ waves through strain gauges pasted on the incident and transmitted bars and based on the assumption of a one-dimensional stress state, while ignoring the friction effect at the interface between the specimen and the bars, the dynamic stress $\sigma (t)$, strain $\varepsilon (t)$, strain rate $\dot{\varepsilon }(t)$, and energy dissipation $W$^41^ of the coal and rock specimens can be calculated using Eqs. (3)–(9):

3 $$ \sigma (t) = \frac{1}{2}E_{0} [\varepsilon_{i} (t) + \varepsilon_{r} (t) + \varepsilon_{t} (t)] $$

4 $$ \varepsilon (t) = \frac{{C_{0} }}{L}\int_{0}^{t} {[\varepsilon_{i} (t) - \varepsilon_{r} (t) - \varepsilon_{t} (t)]} dt $$

5 $$ \dot{\varepsilon }(t) = \frac{{C_{0} }}{L}[\varepsilon_{i} (t) - \varepsilon_{r} (t) - \varepsilon_{t} (t)] $$

6 $$ W_{I} (t) = A_{0} C_{0} E_{0} \int_{0}^{t} {\varepsilon_{i}^{2} } (t)dt $$

7 $$ W_{R} (t) = A_{0} C_{0} E_{0} \int_{0}^{t} {\varepsilon_{r}^{2} } (t)dt $$

8 $$ W_{T} (t) = A_{0} C_{0} E_{0} \int_{0}^{t} {\varepsilon_{t}^{2} } (t)dt $$

9 $$ W = W_{I} (t) - W_{R} (t) - W_{T} (t) $$

In the formulas $W_{I} (t)$, $W_{R} (t)$ and $W_{T} (t)$ represent the incident wave energy, reflected wave energy, and transmitted wave energy, respectively. $A_{0}$, $E_{0}$ and $C_{0}$ are the cross-sectional area, elastic modulus, and wave velocity of the bar, respectively, which are determined values. $L$ is the length of the coal or rock specimen.

Generally, the energy dissipated by coal and rock specimens in SHPB tests includes crushing energy, fragment ejection kinetic energy, thermal energy, acoustic energy, and other types of energy. It is believed that in the case of fracture and crushing with a loading rate that is not particularly high, the dissipation of other types of energy is small and can be neglected. On the one hand, the ejection kinetic energy is difficult to measure. On the other hand, the crushing energy dissipation accounts for more than 95% of the total energy and has a strong linear relationship with the loading rate^62^. For the convenience of the analysis, the dissipated energy of coal and rock was used to approximate the absorbed energy of the specimen^63^. Using Eqs. (6)–(9), the incident energy $W_{I}$, reflected energy $W_{R}$, transmitted energy $W_{T}$, and dissipated energy $W$ during SHPB loading were calculated. Additionally, the energy dissipation density^64^ $\eta_{w}$ and energy conversion efficiency^41^ $k_{w}$ were defined as follows:

10 $$ \eta_{w} { = }W/V $$

11 $$ k_{w} = W/W_{I} $$

where $V$ is the volume of the coal or rock specimen.

#### Box dimensions calculation principle

Research has shown that the microstructure of coal and rock specimens, such as surface cracks, exhibits significant fractal characteristics and that their quantity and distribution characteristics can be quantitatively characterized by fractal dimensions^65^. The fractal dimension can be expressed by the Hausdorff dimension, similar dimension, Kolmogorov capacity dimension, information dimension, correlation dimension, generalized dimension and box dimension^66^. The most commonly used method to describe the fractal dimension of coal and rock cracks is the box dimension method^67^. The basic principle of the two-dimensional box dimension is to cover the surface of coal and rock cracks with a square box with side length $\delta_{i}$ and then count the number of nonempty boxes containing crack elements $N(\delta_{i} )$. By changing the size of the box $\delta$, a series of different $N(\delta )$ values can be obtained^68,69^. Assuming that the surface cracks of coal and rock satisfy fractal theory, the fractal dimension *D* satisfies Eq. (12):

12 $$ N(\delta ) = A\delta^{ - D} $$

where $A$ is the prefactor in the fractal scale rule. For a fixed crack image, $A$ is a constant^70^. Taking the natural logarithm of both sides of Eq. (12), we obtain:

13 $$ \ln N(\delta ) = \ln A - D\ln \delta $$

Equation (13) indicates that by performing linear regression statistics on $\ln \delta$ and $\ln N(\delta )$, the slope of the fitted line is the opposite of the fractal dimension *D*.

Notably, during the testing process, on the one hand, the distinction between coal and rock and cracks is low, and on the other hand, the original images are affected by artefacts, noise, and other noncrack elements recorded by the high-speed camera. In this experiment, Ratsnake software^71^ was used for crack extraction and image annotation, and the FracLac plug-in in ImageJ software^34^ was used to evaluate the box dimensions of the cracks.

#### Mass dimension calculation principle

Similar to surface cracks, fragments of coal and rock under impact also exhibit fractal characteristics over a wide size range^72,73^. However, accurately estimating the number of coal and rock fragments requires a significant amount of work and may even be impossible. In practice, specimen sieves are often used to determine the distribution of fragment sizes after coal and rock impact with the help of the mass‒frequency relationship, which can be expressed as^74,75^:

14 $$ M(x)/M = (x/x_{m} )^{3 - D} $$

where $x$ is the particle size, $x_{m}$ is the average scale, $M(x)$ is the cumulative mass of fragments with a size less than $x$, $M$ is the total mass of the fragments, and $D$ is the fractal dimension of the fragmentation.

Equation (14) shows the relationship between the cumulative mass percentage of coal and rock impact fragments below the sieve and the fractal dimension. Taking the natural logarithm on both sides and rearranging, we obtain:

15 $$ D = 3 - \frac{\ln [M(x)/M]}{{\ln [x/x_{m} ]}} $$

According to Eq. (15), in a coordinate system with the double logarithm of $\ln [x/x_{m} ]$ and $\ln [M(x)/M]$ as the coordinate axes, the data can be linearly fitted, and the slope of the obtained line can then be subtracted from 3 to obtain the fractal dimension *D*.

## Test results and data analysis

### Ultrasonic wave velocity test results for the rock and coal specimens

To facilitate this distinction, the following naming rules were established: C for coal specimens; R for rock specimens; D, FW, SW, and NW for dry, fully saturated water-bearing, semisaturated water-bearing, and naturally water-absorbing specimens, respectively; and FS for fully saturated salt-bearing specimens. Before carrying out the dynamic experiment process, we carried out static loading experiments on different pretreated specimens, as shown in Table 3.

**Table 3:** The mechanical properties of sandstone and coal under static loading conditions.

Specimens	Uniaxial compressive strength (MPa)	Elastic modulus (GPa)
DC	28.98	4.31
FWC	18.24	3.53
FSC	20.02	3.76
SWC	16.37	3.36
NWC	27.77	4.08
DR	38.50	8.37
FWR	23.19	6.82
FSR	25.36	7.34
SWR	19.87	6.76
NWR	31.59	7.78

The ultrasonic longitudinal wave velocity testing was conducted using the ZBL-U5100 nonmetallic ultrasonic detector shown in Fig. 4d, with a transmission voltage of 1000 V and a transmission pulse width of 0.04 ms. The final test results are shown in Table 4.

**Table 4:** P-wave velocity test results for the rock and coal specimens.

Specimen ID	Dry state			Natural water absorption state				Semi saturated water content state				Fully saturated water content state				Saturated saline state			Impact speed (m/s)
	Quality (g)	P wave velocity (km/s)	Average wave velocity (km/s)	Quality (g)	Moisture content (%)	P wave velocity (km/s)	Average wave velocity (km/s)	Quality (g)	Moisture content (%)	P wave velocity (km/s)	Average wave velocity (km/s)	Quality (g)	Moisture content (%)	P wave velocity (km/s)	Average wave velocity (km/s)	Quality (g)	P wave velocity (km/s)	Average wave velocity (km/s)	
DR-1	262.450	1.79	1.94	–	–	–	–	–	–	–	–	–	–	–	–	–	–	–	9.514
DR-2	261.755	2.02		–	–	–	–	–	–	–	–	–	–	–	–	–	–	–	14.925
DR-3	263.290	2.00		–	–	–	–	–	–	–	–	–	–	–	–	–	–	–	17.783
SWR-1	262.754	2.05	1.96	–	–	–	–	263.683	0.35	2.31	2.22	264.605	0.70	2.64	2.52	–	–	–	9.469
SWR-2	262.858	1.90		–	–	–	–	263.685	0.31	2.28		264.390	0.58	2.53		–	–	–	14.018
SWR-3	260.818	1.92		–	–	–	–	261.806	0.38	2.08		262.670	0.71	2.40		–	–	–	18.083
FWR-1	261.449	2.01	1.98	–	–	–	–	–	–	–	–	263.865	0.92	2.66	2.59	–	–	–	9.823
FWR-2	261.040	1.96		–	–	–	–	–	–	–	–	263.343	0.88	2.62		–	–	–	14.204
FWR-3	261.303	1.97		–	–	–	–	–	–	–	–	263.393	0.80	2.48		–	–	–	17.783
FSR-1	261.498	1.98	1.87	–	–	–	–	–	–	–	–	–	–	–	–	263.906	2.78	2.73	9.599
FSR-2	258.949	1.91		–	–	–	–	–	–	–	–	–	–	–	–	261.005	2.75		15.136
FSR-3	265.317	1.71		–	–	–	–	–	–	–	–	–	–	–	–	267.672	2.66		17.647
NWR-1	261.227	1.97	1.93	262.888	0.64	2.11	2.09	–	–	–	–	–	–	–	–	–	–	–	9.914
NWR-2	262.108	1.94		263.750	0.63	2.09		–	–	–	–	–	–	–	–	–	–	–	14.299
NWR-3	261.196	1.88		262.823	0.62	2.07		–	–	–	–	–	–	–	–	–	–	–	17.899
DC-1	138.940	1.17	1.18	–	–	–	–	–	–	–	–	–	–	–	–	–	–	–	5.950
DC-2	143.803	1.12		–	–	–	–	–	–	–	–	–	–	–	–	–	–	–	9.858
DC-3	151.429	1.14		–	–	–	–	–	–	–	–	–	–	–	–	–	–	–	13.823
DC-4	139.605	1.30		–	–	–	–	–	–	–	–	–	–	–	–	–	–	–	17.953
SWC-1	138.505	1.18	1.18	–	–	–	–	139.723	0.88	1.29	1.27	140.983	1.79	1.44	1.43	–	–	–	6.044
SWC-2	155.566	1.17		–	–	–	–	156.316	0.48	1.23		157.151	1.02	1.43		–	–	–	10.298
SWC-3	135.837	1.19		–	–	–	–	136.942	0.81	1.27		138.379	1.87	1.42		–	–	–	14.742
SWC-4	153.958	1.17		–	–	–	–	155.050	0.71	1.28		155.351	0.90	1.43		–	–	–	18.820
FWC-1	142.079	1.11	1.09	–	–	–	–	–	–	–	–	145.207	2.20	1.45	1.47	–	–	–	6.049
FWC-2	136.029	0.91		–	–	–	–	–	–	–	–	139.297	2.40	1.33		–	–	–	9.003
FWC-3	128.184	1.06		–	–	–	–	–	–	–	–	133.329	4.01	1.40		–	–	–	14.734
FWC-4	142.915	1.26		–	–	–	–	–	–	–	–	146.858	2.76	1.71		–	–	–	17.182
FSC-1	140.970	1.16	1.14	–	–	–	–	–	–	–	–	–	–	–	–	143.484	1.74	1.57	6.715
FSC-2	138.892	1.19		–	–	–	–	–	–	–	–	–	–	–	–	140.892	1.74		9.986
FSC-3	115.973	1.13		–	–	–	–	–	–	–	–	–	–	–	–	129.589	1.42		14.713
FSC-4	139.482	1.08		–	–	–	–	–	–	–	–	–	–	–	–	142.032	1.38		18.094
NWC-1	139.014	1.15	1.18	140.139	0.81	1.24	1.21	–	–	–	–	–	–	–	–	–	–	–	6.211
NWC-2	151.76	1.21		152.368	0.40	1.22		–	–	–	–	–	–	–	–	–	–	–	9.157
NWC-3	143.048	1.18		144.502	1.02	1.20		–	–	–	–	–	–	–	–	–	–	–	14.245
NWC-4	139.312	1.17		140.419	0.79	1.18		–	–	–	–	–	–	–	–	–	–	–	18.181

**Figure 6:**
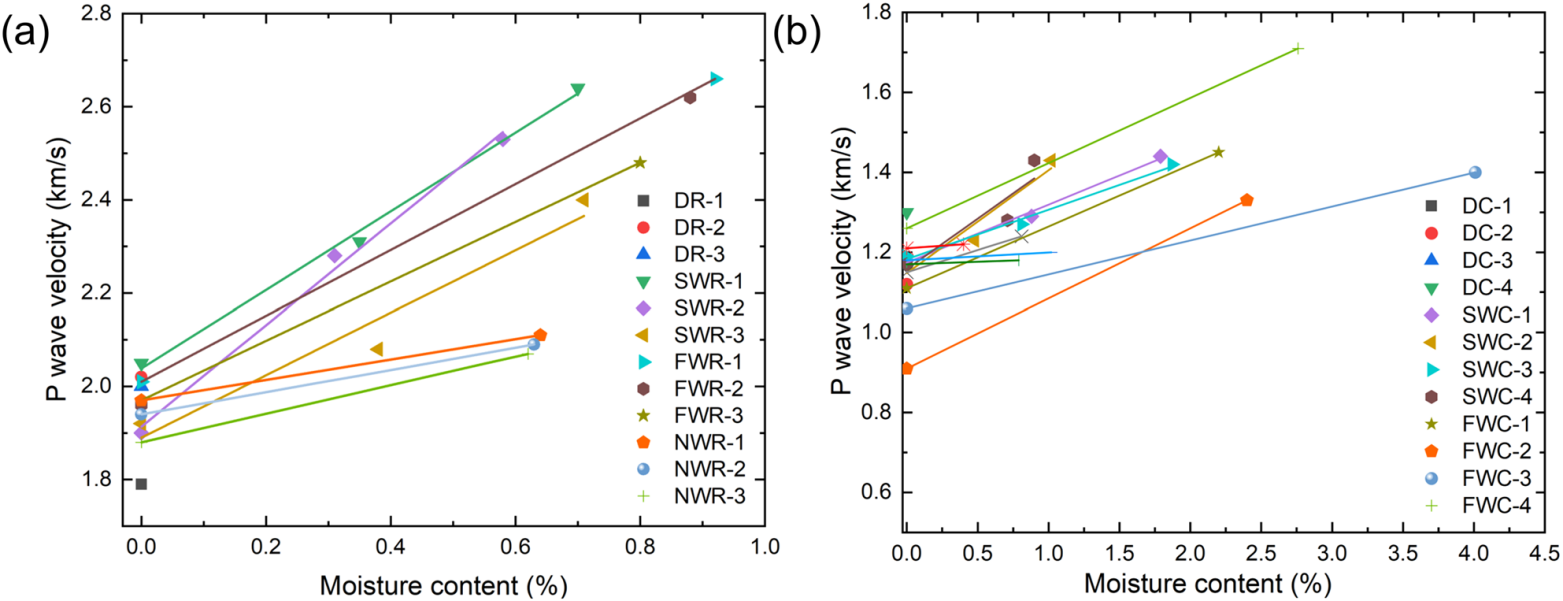
Relationship between the water content and longitudinal wave velocity of the rock and coal specimens. (**a**) Rock specimens; and (**b**) coal specimens.

To investigate the effect of moisture content on the longitudinal wave velocity of the rock and coal specimens, D, NW, SW and FW specimens were selected from Table 4. The scatter plots are shown in Fig. 6 with moisture content $\omega$ as the horizontal axis and longitudinal wave velocity $C_{p}$ as the vertical axis. As shown in Fig. 6, in general, the longitudinal wave speeds of the rock and coal specimens show a typical positive correlation with the water content. This indicates that after the rock and coal specimens absorb water, the invading water molecules effectively fill the fracture space and squeeze the originally existing air, thereby improving the compactness and effective elastic modulus of the specimen. Elastic waves propagate along both the solid skeleton and aqueous medium, which has a better vibration energy transfer property than the solid skeleton and air medium, and the longitudinal wave velocity is effectively increased.

In addition, as shown in Fig. 6a,b, there are some differences in the ultrasonic wave velocity between the coal specimens and rock specimens. For example, (1) in general, the ultrasonic longitudinal wave velocity of the rock specimens is greater than that of the coal specimens. This is particularly significant under dry conditions, indicating that excluding the influence of water, the density or compactness of rock specimens is much greater than that of coal specimens. (2) Linear fitting was performed on the data in Fig. 6a,b, and the final results are shown in Table 5. Comparing the slopes and intercepts of each curve, it was found that the consistency of the slopes of the rock specimens is significantly greater than that of the coal specimens, and the slopes of the specimens under NW conditions are smaller than those under SW conditions. This is because, on the one hand, although the coal and rock specimens used in the experiment do not have obvious structural features, such as joints, bedding, and pores, which significantly affect the longitudinal wave velocity, the anisotropy of the medium and its randomly distributed original structural planes will inevitably affect the acquisition of the wave velocity. On the other hand, saturated water can squeeze pore gas and fill internal spaces more effectively. In comparison, the internal pore space structure of coal specimens is more complex, while the presence of moisture can effectively improve specimen consistency and promote the propagation of ultrasonic waves. During NW absorption, there is still some air in the specimen, and the interior of the specimen is actually in a state where multiple phases of solid, liquid, and gas coexist. A random distribution of media with different densities directly leads to an increase in the probability of elastic wave reflection, refraction, and diffraction during propagation, ‘extending’ the propagation path of waves and making the increase in wave velocity slower^76^. As the moisture content in the specimen increases, the specimen gradually transforms into a ‘solid‒liquid coupled body’ of a coal or rock skeleton and water, and the longitudinal wave propagates through the coupled body, with a more significant increase in longitudinal wave velocity.

**Table 5:** Experimental results of the water content and longitudinal wave velocity of coal and rock.

Specimen ID	Wave velocity of dried specimen (km/s)	Fitting curve *y* = a + b*ω*		Specimen ID	Wave velocity of dried specimen (km/s)	Fitting curve *y* = a + b*ω*	
		a	b			a	b
DR-1	1.79	–	–	DC-1	1.17	–	–
DR-2	2.02	–	–	DC-2	1.12	–	–
DR-3	2.00	–	–	DC-3	1.14	–	–
SWR-1	2.05	2.04	0.84	DC-4	1.30	–	–
SWR-2	1.90	1.91	1.09	SWC-1	1.18	1.17	0.15
SWR-3	1.92	1.89	0.67	SWC-2	1.17	1.15	0.26
FWR-1	2.01	2.01	0.71	SWC-3	1.19	1.18	0.12
FWR-2	1.96	1.96	0.75	SWC-4	1.17	1.16	0.25
FWR-3	1.97	1.97	0.64	FWC-1	1.11	1.11	0.15
NWR-1	1.97	1.97	0.22	FWC-2	0.91	0.91	0.18
NWR-2	1.94	1.94	0.24	FWC-3	1.06	1.06	0.08
NWR-3	1.88	1.88	0.31	FWC-4	1.26	1.26	0.16
				NWC-1	1.15	1.15	0.11
				NWC-2	1.21	1.21	0.03
				NWC-3	1.18	1.18	0.02
				NWC-4	1.17	1.17	0.01

As shown in Table 5, under the same soaking conditions, the ultrasonic wave velocity of the saturated salt-containing specimens is greater than that of the saturated water-containing specimens. This indicates that the presence of Na^+^ and Cl^−^ ions plays a certain role in promoting the entry of water molecules into coal and rock pores. Due to the diverse mineral composition and complex structural surfaces of coal and rock, further clarification is needed on the physical and chemical effects of solutions on solids^77^. It is speculated that the influence of the solution on the ultrasonic wave velocity mainly involves two aspects: (1) the ions in the solution react physically and chemically with the coal and rock, promoting the dissolution of the solid skeleton and ion exchange and increasing the porosity of the specimen^78^. (2) The presence of ions promotes capillary phenomena, which helps liquids quickly fill solid pores^79^, resulting in an enhanced trend in the overall wave velocity of specimens. Obviously, for the coal and rock specimens used in the experiment, the latter is more dominant, which determines the final result. Notably, the correlation analysis between the ultrasonic wave velocity and water content in this section is based on individual specimens. For a fixed specimen, the porosity and thus the saturated water content are fixed, which provides a result different from the result obtained by selecting different specimens as research objects for statistical analysis^80,81^.

### Dynamic response of the rock and coal specimens

#### Dynamic stress equilibrium

**Figure 7:**
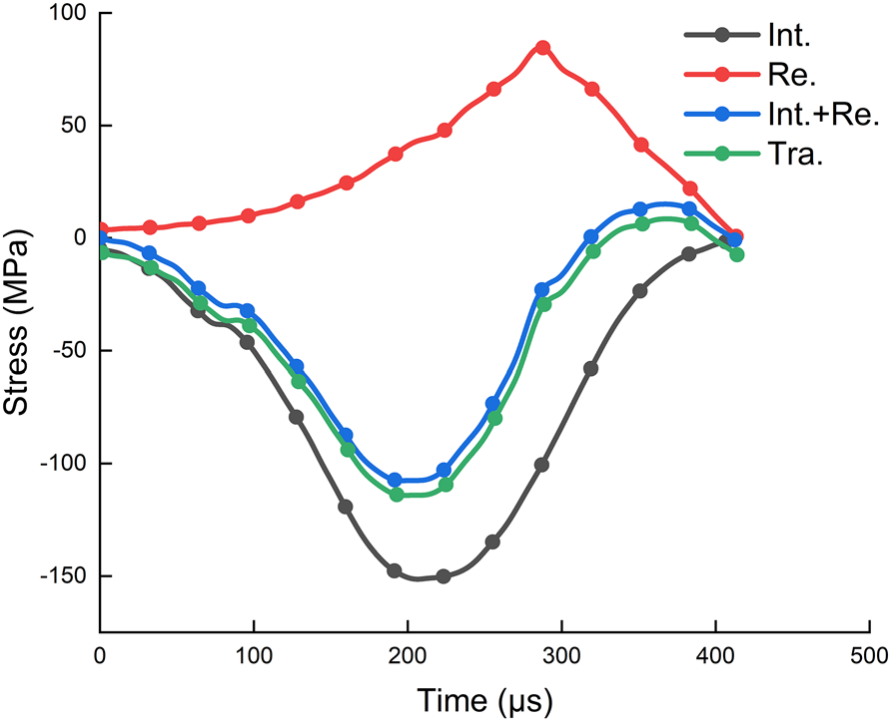
Stress balance inspection of a specimen (Int: incident wave, Re: reflected wave; and Tra: transmitted wave).

Checking the stress balance of the specimen is a prerequisite for ensuring the accuracy of the SHPB test results^41^. After each impact experiment, it is necessary to first check the mechanical equilibrium at both ends of the specimen. Figure 7 shows the processing results of a typical experiment, which was obtained from a natural water-absorbing rock specimen (NWR-1) with an impact velocity of 9.914 m/s. According to the test signals on the elastic bars, the stress at both ends of the specimen can be obtained. Specifically, the incident stress (Int + Re) is obtained by superimposing the incident wave and reflected wave, and the transmitted stress (Tra) is obtained from the transmitted wave. Obviously, in Fig. 7, the two curves are basically coincident. Therefore, the forces at both ends of the specimen are basically in a dynamic equilibrium throughout the process from deformation to destruction, which provides strong support for ensuring the scientificity and accuracy of subsequent test results.

#### Stress–strain curves

**Figure 8:**
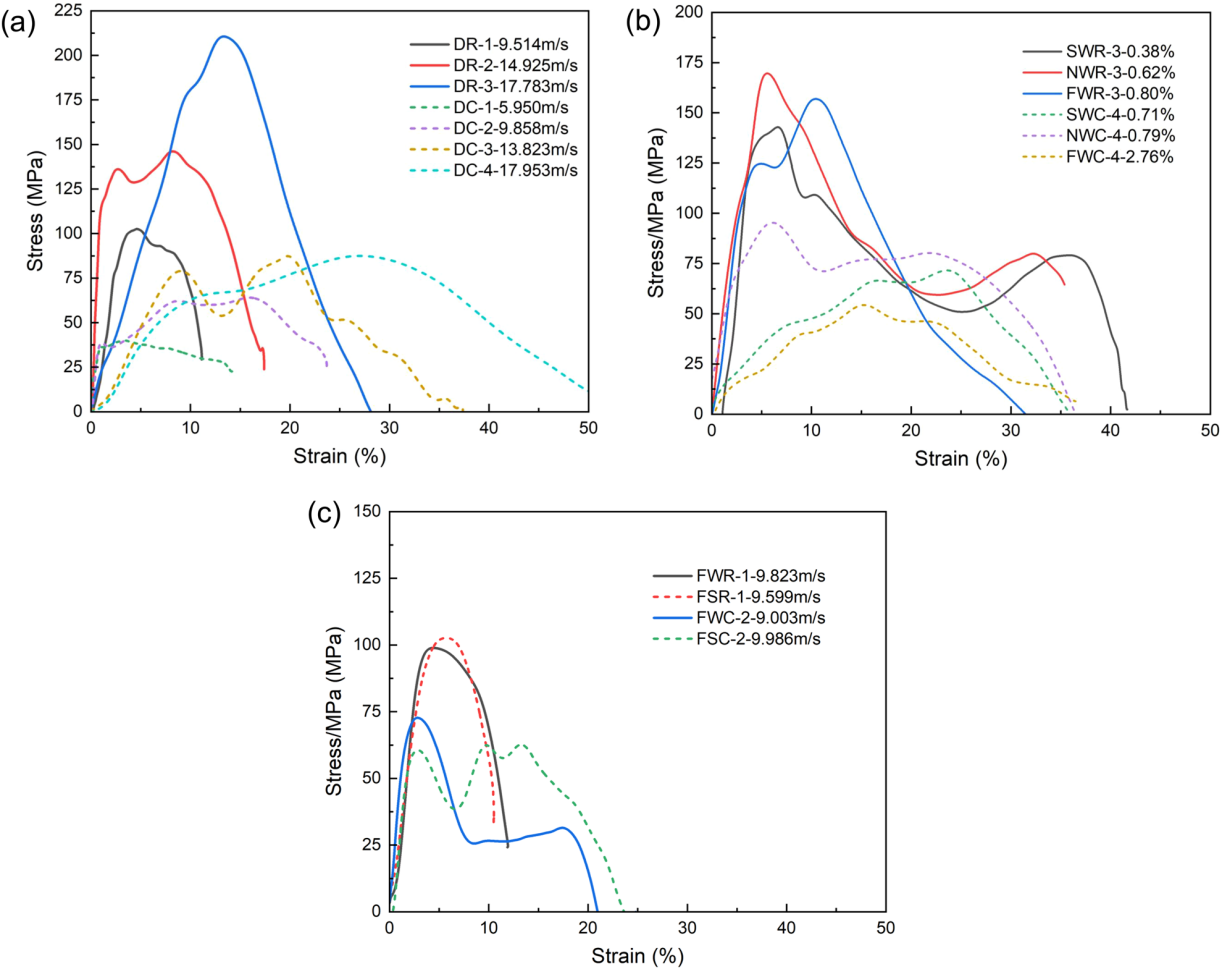
Influence of different factors on the dynamic stress‒strain curves. (**a**) Impact velocity (*ω* = 0%), (**b**) moisture content (v ≈ 18 m/s), and (**c**) distilled water immersion and saline immersion (v ≈ 9 m/s).

To effectively reveal the trend of the stress wave variation in coal and rock during the SHPB impact process, the recorded signals from the semiconductor strain gauges attached to the input and output bars were extracted for analysis. Using Eqs. (3)–(5), the influence of impact velocity, moisture content, and saturated saltwater on the dynamic stress‒strain relationship of the coal and rock specimens was investigated, and the curves were plotted, as shown in Fig. 8.

Figure 8a shows the effect of impact velocity on the dynamic uniaxial compressive strength, failure strain, and dynamic elastic modulus of coal and rock specimens under dry (moisture content *ω* = 0) conditions. As shown in the figure, the stress‒strain curves of each specimen exhibit both consistency and variability. This consistency is manifested as follows: (1) Each stress‒strain curve has distinct stage characteristics, and all undergo linear elastic deformation, strengthening, and strain softening stages, in which the strengthening stage is an important period for the initiation and propagation of tip cracks^34^. (2) As the impact velocity increases, the strain strengthening effect of the coal and rock materials gradually becomes prominent, and the peak stress increases accordingly. (3) Due to the heterogeneity of the coal and rock materials, the variation trend of the dynamic elastic modulus of the test specimens does not exhibit a high degree of consistency with the impact velocity (i.e., strain rate). In comparison, the dynamic elastic modulus is less sensitive to the strain rate, exhibiting a strain rate passivation characteristic. In addition, the stress‒strain curves of coal and rock also exhibit some differences, mainly as follows: (1) compared with rock specimens, coal specimens have a shorter overall elastic rise phase, a longer plastic deformation phase, and greater fluctuations in stress. (2) The peak stress of the coal specimens is significantly lower than that of the rock specimens, while the failure strain is significantly greater than that of the rock specimens. The results indicate that although both are elastic‒plastic materials, rocks exhibit more elastic characteristics, and plastic characteristics are more evident in coal.

As shown in Fig. 8b, there are significant differences in the dynamic mechanical properties of coal and rock specimens with different moisture contents under an impact velocity of 18 m/s. Specifically, (1) the peak stress of rock specimens does not show a monotonic increase or decrease with increasing moisture content. The NWR with a moisture content of 0.62% has the highest dynamic compressive strength, followed by the FSR with a moisture content of 0.80%, while the SWR with a moisture content of 0.38% has the lowest dynamic compressive strength but the maximum failure strain. (2) With increasing moisture content, both the dynamic compressive strength and dynamic elastic modulus of the coal specimens decrease, and the peak strain increases accordingly. This indicates that compared to those of rocks, the dynamic mechanical properties and strength characteristics of coal bodies are more sensitive to changes in moisture content. The reason for this is that water has two effects on the dynamic mechanics of coal and rock: on the one hand, water dissolves the structure of the coal and rock, making the connection between the particles loose and the structure porous^82^. In other words, as the moisture content increases, the interconnectivity between weaker pores and cracks in the specimen increases, and water expands from the surface of the specimen into the interior, deteriorating the physical and mechanical state of the coal and rock and weakening the dynamic mechanical properties of the specimen. On the other hand, under high impact velocity conditions, cracks rapidly expand in the vertical direction, and the surface tension, internal friction, and viscosity (Stefan effect) of water increase the resistance to crack expansion, thereby enhancing the dynamic uniaxial compressive strength of the specimen^83,84^. Obviously, due to the more developed primary fractures and greater porosity of coal specimens, water can easily be absorbed, increasing susceptibility to the effects of water erosion. In summary, water has a weakening effect on the physical and mechanical properties of coal specimens overall. However, further research is needed on the influence of water on rock specimens, including the impact velocity and original characteristics of the specimens.

The stress‒strain curves of the coal and rock specimens under similar impact velocities (v ≈ 9 m/s) and under the same pretreatment method (complete immersion) were collated, as shown in Fig. 8c. As shown in the figure, similar to Fig. 8b, due to the differences in the coal-rock pores and solid skeleton, the influence of the immersion medium on the coal is much greater than that on the rock. Compared with those of the specimens immersed in distilled water, the uniaxial compressive strength of the specimens immersed in saltwater is 16.1% lower, the elastic modulus is 3.18% lower, and the peak strain is 1.5% greater. Therefore, the weakening effect of saltwater on the physical mechanics of coal is much greater than that of distilled water. After saltwater erosion, the elastic stage of the specimen shortened, and the plastic deformation stage extended, indicating that the brittleness of the specimen decreased and that the ductility of the specimen increased.

#### Energy dissipation characteristics

**Figure 9:**
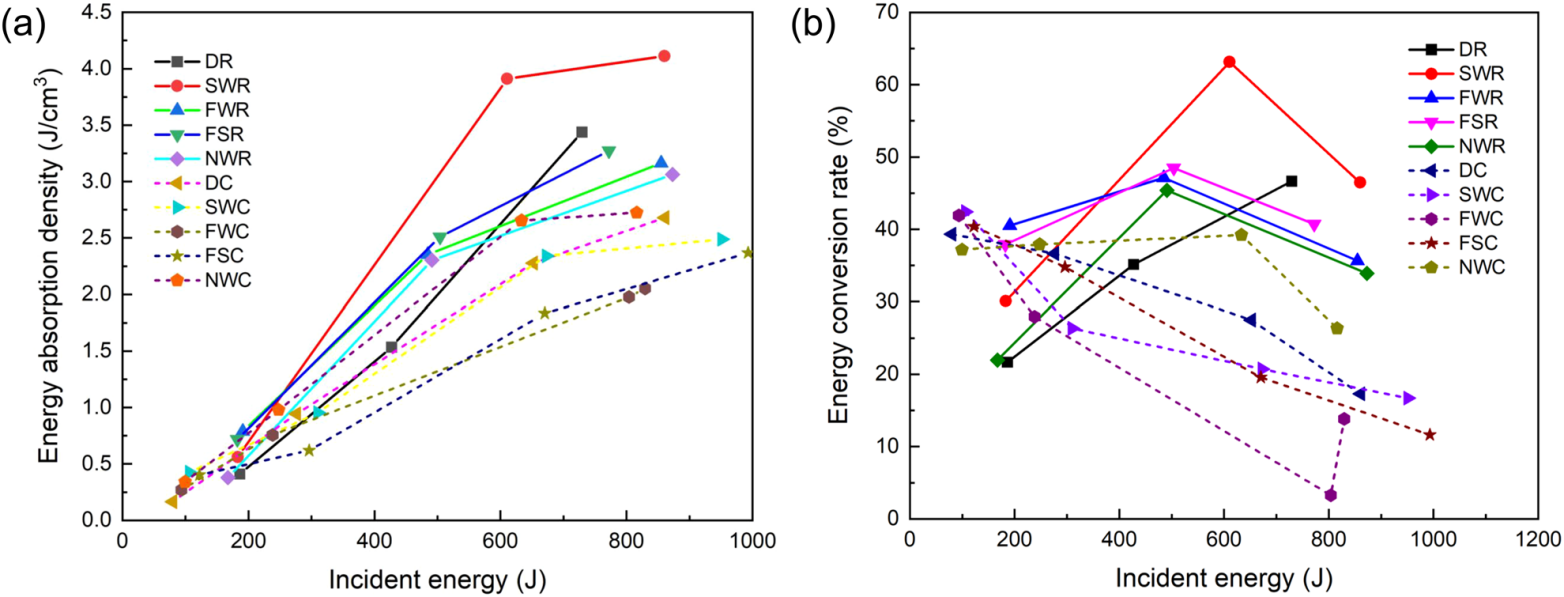
Summary of experimental results on the effect of incident energy on the energy dissipation density and energy conversion rate of coal or rock materials. (**a**) Energy dissipation density versus incident energy and (**b**) energy conversion rate versus incident energy.

Based on Eqs. (10)–(11), the relationships between $\eta_{w}$, $k_{w}$, and the incident energy $W$ are plotted in Fig. 9.

In Fig. 9a, the rock and coal specimens exhibit similar patterns; that is, with increasing incident wave energy, the energy dissipated per unit volume also increases synchronously, and both exhibit a strong linear correlation. In addition, the above curves show some differences: (1) Under similar incident wave energy conditions, the energy dissipation density of rock is generally greater than that of coal. The greater the impact velocity is, the more significant the difference between the two. (2) When the incident energy is low (the impact velocity is 9 m/s), the energy dissipation efficiency of rock and coal immersed in saltwater is slightly lower than that of rock and coal immersed in distilled water. With increasing impact velocity, the former is greater than the latter. This is because under low-incident energy conditions, saltwater has a greater erosion effect on the coal and rock matrix than does distilled water, and the specimens immersed in saltwater are subjected to more damage before impact^47^. In contrast, because saltwater is more prone to invade the interior of coal and rock bodies, under high-speed impact conditions, the surface tension and Stefan effect of water, which are required to overcome crack initiation and propagation, will also increase, resulting in an increase in the energy dissipation of the specimen.

Figure 9b shows the relationship between the energy conversion efficiency and incident energy. It is evident that rock and coal exhibit distinct characteristics. For coal, the energy conversion efficiency of the specimen decreases with increasing incident energy. Correspondingly, the energy conversion efficiency of rock specimens often tends to first increase and then decrease, resulting in a peak. This indicates that when the incident energy is low, the energy absorbed by the specimen is basically used for specimen fracture development. When the incident energy is very high, on the one hand, part of the energy will be converted into kinetic energy of the specimen fragments sputtering out (which is not reflected in Eq. 17); on the other hand, a large amount of energy will be retransferred back to the bars in the form of reflected or transmitted energy. Although both are elastic‒plastic bodies, the brittleness of rocks is more pronounced than that of coal, and the production of more ejected fragments consumes more elastic energy. Moreover, rocks have better energy transfer properties, which together lead to the existence of an inflection point in the energy conversion efficiency curve for rock specimens. For coals, its outstanding ductility reduces the proportion of ejected energy; therefore, it is more difficult for energy to be transmitted to the bars. Therefore, the coal specimens exhibit a monotonic decreasing trend of energy adsorption, and the energy absorbed by the coal is used more for generating new surfaces and structural damage. Notably, compared with coal and rock specimens immersed in distilled water, specimens immersed in saltwater achieve a higher energy conversion rate, indicating that the latter plays a promoting role in increasing the proportion of surface energy in the overall energy conversion.

### Failure mode and quantitative characterization of surface cracks

**Figure 10:**
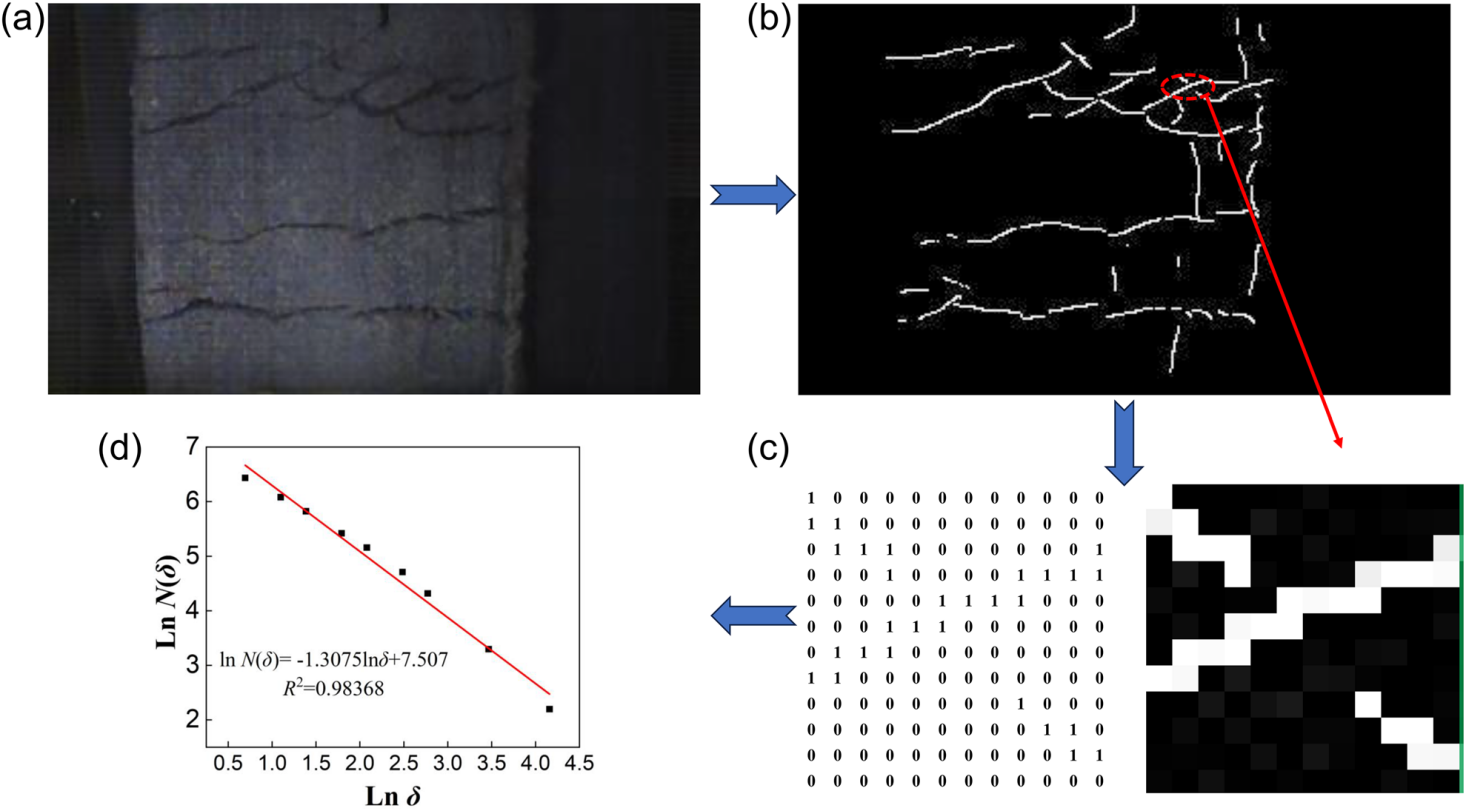
Calculation process of the fractal dimension of the NRW-3 specimen at 160 μs. (**a**) Original image; (**b**) binary extraction results using Ratsnake software; (**c**) box coverage and nonempty box counting; and (**d**) regression analysis and fractal dimension calculation.

In the quantitative study of surface cracks during coal and rock failure, the box dimension method is most widely used^85–87^, therefore this method was also adopted in this article. Due to the low contrast between the recorded surface cracks and the specimen in this experiment, the full information of the cracks cannot be completely reflected. Therefore, Ratsnake software and the FracLac plugin loaded on ImageJ software were used to achieve quantitative characterization of surface cracks in coal and rock specimens^34^. The basic process is shown in Fig. 10.

As shown in Fig. 10a, on the one hand, affected by imaging equipment and external environmental interference, the original images obtained using high-speed cameras contain a large amount of noise, which affects the intuitive judgement of the experimental results. On the other hand, the cracks also exhibit the typical characteristics of relatively small target width, low image resolution and contrast, and poor crack continuity, with bifurcations and stray points, which pose challenges for the feature extraction and quantitative characterization of cracks. To this end, in this article, Ratsnake annotation software was used to extract and label the cracks in the images at the pixel level^88,89^. The results are shown in Fig. 10b, which clearly displays the details of the crack images. As shown in Fig. 10c, while completing image segmentation and morphological processing, Ratsnake software also converts the colour mode of the image from an RGB true-colour image to a binary image that is suitable for subsequent rapid analysis. Specifically, crack elements are highlighted and defined as white, while noncrack backgrounds are defined as black. Subsequently, based on the fundamental principle of box dimension, the number of nonempty boxes containing crack elements is counted, and the result is presented in the form of a two-dimensional matrix. Obviously, the number of statistical values is closely related to the size of the boxes, and both satisfy the functional relationship shown in Eq. (13) in the previous section. Using the FracLac plug-in embedded in ImageJ software to process the crack image shown in Fig. 10b, the numbers of nonempty boxes under 9 different grid sizes were counted separately, and the logarithmic relationship between box size and count data was plotted as a scatter plot, as shown in Fig. 10d. Figure 10d reveals that the fitting exhibits a good linear correlation, with a fit coefficient of 0.98, in which case the regression analysis yielded a fractal dimension of 1.3075.

**Figure 11:**
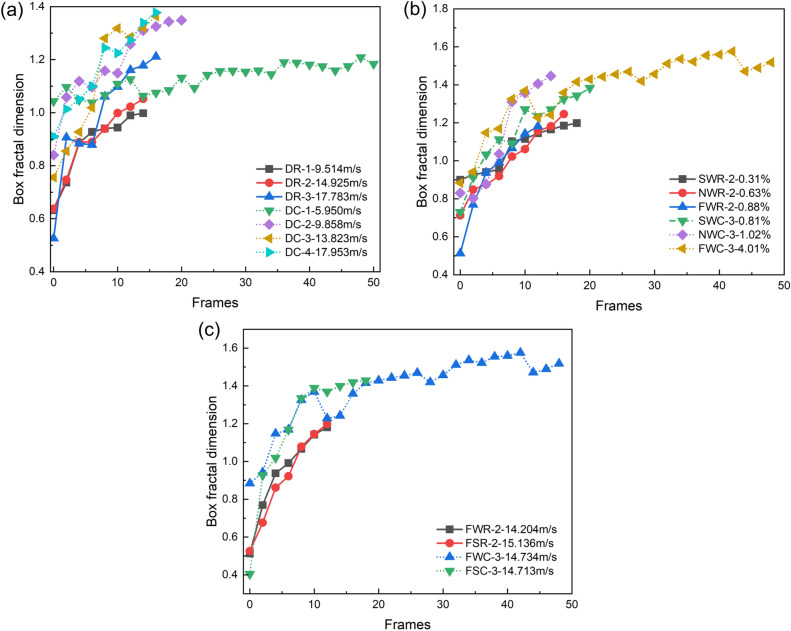
Influence of different factors on the box fractal dimension. (**a**) Box dimension versus velocity; (**b**) box dimension versus moisture content; and (**c**) box dimension versus saltwater and distilled water immersion.

The calculation process of the box dimension at 140 μs for the NRW-3 specimen was detailed in the previous section as an example. Similarly, the same processing was performed on the crack images of other coal and rock specimens to calculate the entire process from crack initiation to fragment ejection during the SHPB impact experiment. The image frame frequency of the high-speed camera was 100,000 FPS, and one image was selected at an interval of 20 μs, resulting in a total of 395 valid images being extracted. Due to space limitations, only some of the results are shown in Fig. 11.

Figure 11a demonstrates the effect of impact velocity on the evolution of surface cracks in dry coal and rock specimens. When the water content and other related factors are not considered, the box dimensions of both the rock and coal specimens exhibit the remarkable characteristics of overall increase and local oscillation. As the impact velocity increases, the box dimension values of each specimen increase simultaneously, and the cut-off time of the curve also decreases, indicating that the speed, opening, and final degree of surface crack propagation in the specimen increase with increasing strain rate, and the evolutioary process also significantly accelerates. This is reflected in both the rock and coal specimens. However, there are some differences in the box dimension changes between coal and rock at similar impact velocities. Specifically, compared with those of rock, the box dimension curves of dry coal exhibit higher starting values, lower initial slope values, larger final values, and wider range characteristics of the box dimension, indicating that although both are elastic‒plastic bodies with heterogeneous, discontinuous, and randomly distributed material parameters, the plasticity of coal is significantly greater than that of rock. In contrast, the rock exhibits more brittle characteristics.

Figure 11b shows the correlation between the moisture content and surface crack evolution at an impact rate of 14 m/s. The figure shows that as the moisture content increases, the degradation effect of water on the solid matrix becomes more pronounced, and the final value of the box dimension of the surface cracks after impact on the specimen increases accordingly. Notably, specimens with higher moisture contents exhibit two interesting characteristics under impact: (1) the range of the box dimension curve increases significantly, the plastic stage grows, and the damage process of the specimen is significantly delayed; and (2) in the initial stage of impact, the box dimension of surface cracks is relatively low. This characteristic does not change until the opening of the main crack increases to a certain extent. Obviously, under high strain rates, crack propagation speeds are faster, and mechanical factors such as the Stefan effect and Newtonian internal friction effect of free water in coal and rock masses will occur, resulting in resistance to delayed crack propagation^83,84^. Compared with those of rock, these characteristics are more prominent in coal specimens.

In Fig. 11c, because the corrosion and dissolution of saline water on solid substrates are greater than those of distilled water, at similar dynamic impact velocities, the box fractal dimension of surface cracks in coal and rock specimens immersed in saline water is greater than that in coal and rock specimens immersed in distilled water, and the cut-off time of the box dimension curve is advanced. Similarly, this characteristic is more prominent in coal body specimens.

### Fragmentation effect and mass fractal dimension

**Figure 12:**
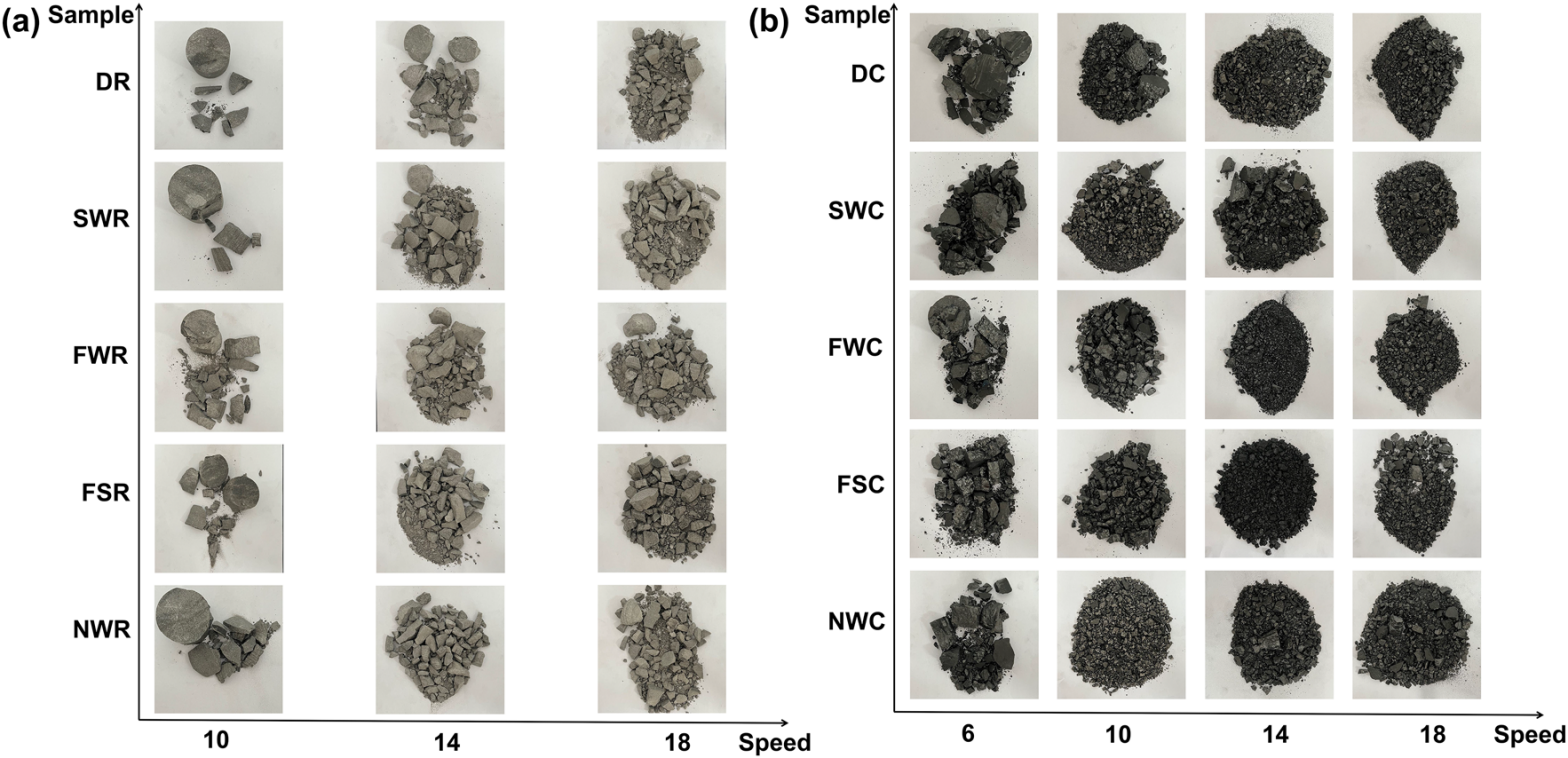
Failure modes of the coal and rock specimens. (**a**) Rock and (**b**) coal.

Notably, the variation trend of the box dimension can only reflect the characteristics of the crack evolutionary process during coal and rock damage from the surface and local areas, and it is difficult to systematically and comprehensively explore the crushing effect of specimens after dynamic load impact. In view of this, fragments of coal and rock specimens after impact damage were collected, as shown in Fig. 12. When the impact velocity is low, the incident energy is low, and the coal and rock specimens are less damaged, resulting in larger fragment sizes. When the impact velocity is low, the specimens (especially the rock specimens) exhibit only the peeling of smaller blocks. As the impact velocity increases, the average fragment size of the coal and rock crushing bodies continues to decrease, the number of fragments continues to increase, the crushing degree continues to increase, and a large amount of powder forms. The failure mode of the specimens transforms from splitting to crushing to smashing.

**Figure 13:**
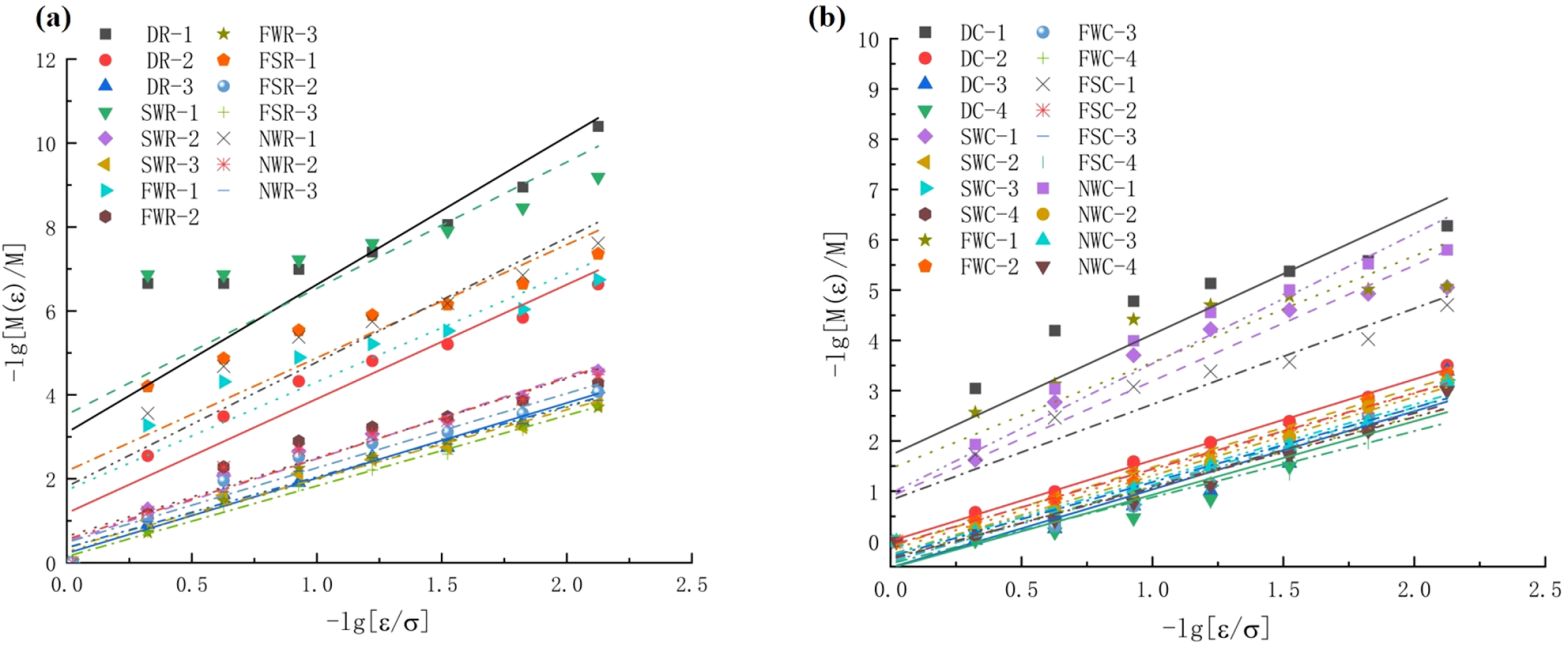
Statistical chart of the cumulative particle size distribution. (**a**) Rock and (**b**) coal.

Based on the qualitative analysis above, the fragmented blocks were screened and weighed by the specimen-splitting sieve shown in Fig. 4e to quantitatively summarize the distribution of different size-based fragmented block structures. Fractal analysis of the fragments was conducted based on Eq. (15), and the calculated fractal dimensions are shown in Fig. 13 and Table 6.

**Table 6:** Calculation results for the mass fractal dimension.

Specimen no	Impact speed (m/s)	Cumulative mass percent of each particle size under the sieve(%)								Fitting results		Fractal dimension
		0.075 mm	0.15 mm	0.30 mm	0.60 mm	1.18 mm	2.36 mm	4.75 mm	9.50 mm	Fitting slope	Decision factor (*R*^2^ Value)	
DR-1	9.514	–	–	–	–	–	–	–	–	–	–	–
DR-2	14.925	0.131	0.290	0.547	0.815	1.323	3.066	7.837	100	2.724	0.909	0.277
DR-3	17.783	1.599	3.497	6.435	7.956	14.842	19.697	40.251	100	1.792	0.980	1.208
SWR-1	9.469	–	–	–	–	–	–	–	–	–	–	–
SWR-2	14.018	1.043	1.955	3.362	4.645	7.037	12.570	27.836	100	1.969	0.960	1.031
SWR-3	18.083	2.239	3.933	6.416	8.552	12.006	20.109	36.466	100	1.647	0.971	1.353
FWR-1	9.823	0.117	0.239	0.398	0.545	0.751	1.338	3.779	100	2.581	0.815	0.419
FWR-2	14.204	1.404	2.128	3.084	3.957	5.519	10.215	31.585	100	1.877	0.916	1.123
FWR-3	17.783	2.453	3.652	5.452	8.136	10.559	22.926	48.465	100	1.725	0.970	1.275
FSR-1	9.599	0.064	0.130	0.213	0.274	0.394	0.770	1.496	100	2.699	0.759	0.301
FSR-2	15.136	1.706	2.853	4.439	5.804	8.083	14.176	34.196	100	1.776	0.948	1.224
FSR-3	17.647	2.366	4.233	7.525	10.974	16.011	25.343	47.785	100	1.681	0.991	1.319
NWR-1	9.914	0.049	0.106	0.207	0.320	0.463	0.927	2.845	100	2.964	0.840	0.036
NWR-2	14.299	1.134	2.038	3.372	4.622	6.755	10.324	30.079	100	1.927	0.944	1.073
NWR-3	17.899	2.080	3.629	5.908	8.211	11.218	18.456	37.436	100	1.687	0.969	1.313
DC-1	5.950	0.188	0.376	0.463	0.589	0.839	1.507	4.762	100	2.404	0.788	0.596
DC-2	9.858	3.010	5.671	9.187	13.944	20.451	37.095	55.994	100	1.609	0.997	1.391
DC-3	13.823	3.372	10.333	21.061	36.384	49.213	77.586	97.466	100	1.554	0.909	1.446
DC-4	17.953	4.594	10.874	22.456	43.111	62.591	82.627	97.680	100	1.461	0.900	1.539
SWC-1	6.044	0.639	0.723	1.001	1.465	2.462	6.254	19.686	100	2.299	0.892	0.701
SWC-2	10.298	3.561	5.851	11.259	17.656	25.032	42.488	65.793	100	1.578	0.996	1.422
SWC-3	14.742	4.023	8.674	15.230	32.828	48.025	65.272	86.345	100	1.536	0.947	1.464
SWC-4	18.820	4.509	10.959	16.128	31.946	43.952	66.348	87.919	100	1.455	0.955	1.545
FWC-1	6.049	0.628	0.663	0.762	0.906	1.207	4.374	7.671	100	2.115	0.775	0.885
FWC-2	9.003	3.549	6.237	13.050	22.560	30.801	43.248	67.225	100	1.554	0.982	1.446
FWC-3	14.734	4.579	8.173	17.378	31.777	49.598	73.688	81.907	100	1.503	0.944	1.497
FWC-4	17.182	4.913	11.100	19.078	30.549	42.081	63.267	87.254	100	1.401	0.961	1.599
FSC-1	6.715	0.906	1.782	2.815	3.386	4.610	8.486	17.726	100	1.907	0.904	1.093
FSC-2	9.986	3.941	7.816	11.026	15.503	24.564	45.607	68.424	100	1.516	0.994	1.484
FSC-3	14.713	4.888	8.751	16.241	22.927	36.181	62.140	90.921	100	1.480	0.982	1.520
FSC-4	18.094	5.499	14.207	26.372	46.151	59.742	74.905	95.523	100	1.318	0.897	1.682
NWC-1	6.211	0.303	0.399	0.671	1.052	1.843	4.796	14.491	100	2.581	0.916	0.419
NWC-2	9.157	4.139	6.848	12.337	20.724	36.692	54.796	77.147	100	1.565	0.986	1.435
NWC-3	14.245	4.165	9.195	15.005	22.437	34.918	58.811	82.417	100	1.498	0.980	1.502
NWC-4	18.181	4.975	11.022	18.157	32.821	46.268	65.190	85.701	100	1.406	0.955	1.594

Comparing Fig. 13 and Table 6, it can be seen that the *R*^2^ index of the fitted cumulative mass percentage of each particle size below the sieve and the fitted size of the specimen sieve aperture are between 0.759 and 0.997, indicating a good logarithmic linear correlation between the two. Notably, because DR-1 and SWR-1 only broke into a limited number of larger pieces after impact, their fragment screening results were not significant; therefore, these cases were discarded during analysis. Table 6 also shows the following findings: (1) The change in the strain rate plays a key role in the mass frequency distribution of the specimen damage products. As the strain rate increases, the specimen damage-induced fragment size gradually decreases, and the mass fractal dimension simultaneously increases. Taking the dry state as an example, the mass fractal dimensions of coal and rock increase from 0.277 and 0.596 to 1.208 and 1.539, respectively. At the same time, under similar impact velocities, the mass fractal dimension of coal also far exceeds that of rock, indicating that the damage degree of coal specimens is more severe than that of rock specimens. (2) Generally, with increasing moisture content, the damage degree of the specimen increases, and the bulk ratio of the specimen after crushing gradually decreases. The mass fractal dimension generally shows a trend of FW > SW > NW. (3) At similar impact velocities, specimens immersed in saltwater have a slightly lower ability to resist crushing than those immersed in distilled water, and the final degree of damage to the specimen is often greater.

Therefore, the mass fractal dimension can effectively reflect the fragment size distribution characteristics of coal and rock fragments, and the damage morphology of specimens is affected by both external impact loads and the internal physical and mechanical properties of the material.

## Discussion

### Microscopic characterization of crack evolution and failure modes under the coupled effect of saltwater and dynamics

**Figure 14:**
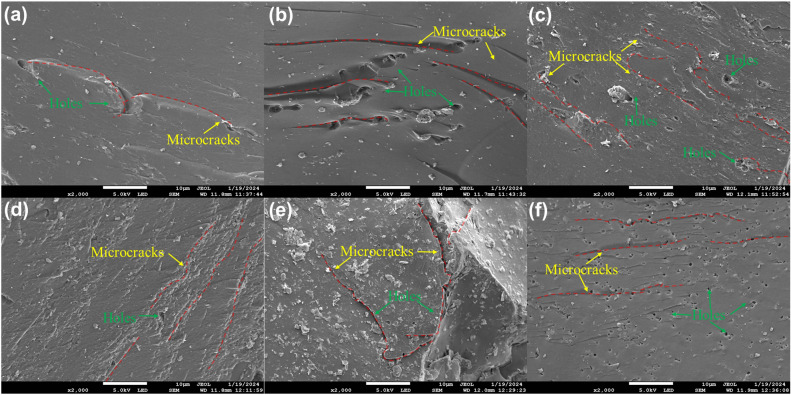
SEM images of coal and rock at an impact velocity of 14 m/s. (**a**) DC-3; (**b**) FWC-3; (**c**) FSC-3; (**d**) DR-2; (**e**) FWR-2; and (**f**) FSR-2.

Coal and rock contain many pores, fractures, and gas‒liquid solid multiphase coupling and other complex heterogeneous structures^90^. These microstructures play an important role in determining the macroscopic mechanical behaviour and failure characteristics of the specimen^91^. In addition, distilled water and saline solution can have a significant impact on the microstructure of coal and rock^92^. In summary, characterizing the internal structure and unstable crack propagation characteristics of coal and rock impact failure in a specimen under the action of distilled water and saline solution at the microscopic scale will aid in understanding the failure mechanism of the specimen. Therefore, with the help of a JSM-7900F in situ variable vacuum field emission scanning electron microscope at the State Key Laboratory of Clean and Efficient Coal Utilization at Taiyuan University of Technology, SEM analysis was carried out on coal and rock microsections after impact failure under different conditions, as shown in Fig. 14.

Figure 14a–c shows microscopic surface electron micrographs of the coal specimens under dry, distilled water, and saturated saltwater immersion conditions. In the dry state, the internal structure of the coal specimens is relatively clear and smooth, with only a few microcracks and pores. The cracks show a herringbone pattern. There are two possible reasons for this type of cracking: (1) When the cracks propagate, they preferentially follow the path of least resistance, i.e., along the holes present in the coal specimens. When two cracks converge into the same pore, a herringbone pattern is formed^93^. (2) As a primary defect, the pores in Fig. 14a can be regarded as the ‘initiation source’ of cracks. Under high-speed impact, stress cannot instantaneously propagate and distribute to distant locations from the ‘initiation source’ and can only play a role in the vicinity. When the stress near the crack tip exceeds the fracture toughness of coal, tensile cracks begin to gradually expand from the crack tip to both ends. Therefore, the development trend of herringbone patterns can effectively determine the propagation mode of cracks^94^.

Figure 14b shows the SEM image of coal specimen fragments after being immersed in distilled water and damaged by impact. Compared with dry specimens, the number of internal microcracks gradually increases, and the connectivity between them is enhanced. The cracks mainly show step-like and river-like patterns, which are formed by the breaking of the bonds between cleavage planes. Clearly, this indicates that the integrity of the coal specimen has deteriorated to some extent. After the coal specimen is immersed in saltwater, the specimen undergoes hydration, with debris particles attached^46^. The surface becomes rough and uneven, and larger-volume pores appear, as shown in Fig. 14c. This indicates that the internal structure of the coal specimen has undergone significant loosening and further deterioration. On the one hand, both water and brine dissolve some substances in the coal specimen, gradually increasing its internal pores. On the other hand, water molecules invade the cracks in the coal specimen, exacerbating microcrack growth and expansion and reducing the stability of the coal specimen, and the degradation effect of brine on the coal specimen becomes more pronounced^95^.

Figure 14d–f shows electron microscopy images of the microscopic surfaces of the rock specimens under dry, distilled water immersion, and saturated saltwater immersion conditions, respectively. The figures show that there are both similarities and differences between the rock specimens and coal specimens. The number of microscopic surface pores and fissures in both the rock specimens and coal specimens decrease in the following order: dryness < distilled water immersion < saltwater immersion. However, regardless of the state, there are fewer microscopic surface fissures and pores in the rock specimen than in the coal specimen, and the smoothness of a rock specimen is also greater than that of the corresponding coal specimen. In particular, comparing Fig. 14c,f, although there are also many pores due to the deterioration from the saltwater, the sizes of the pores in the rock specimen are much smaller than those of the coal specimen, and the interparticle cementation is much stronger than that of the coal specimen; therefore, the rock specimen does not produce intricate fissures similar to those of the coal specimen. Therefore, the resistance of the rock specimens to water and brine deterioration is greater than that of the coal specimens.

### The influential mechanism of distilled water and saline water on dynamic effects

Both coal and rock are extremely complex heterogeneous anisotropic porous media, with their main physical components being the matrix, void space, and minerals^96^. The matrix contains organic components, while minerals are mainly inorganic components. Void space refers to the volume occupied by pores and cracks and can provide channels for water or aqueous solutions of different natures to enter the interior of coal and rock. The water or aqueous solution infiltrating coal and rock undergo water–rock interactions (WRIs) with the solid components, which involve multifield coupling of fields such as the stress field, fracture field, and seepage field, and many physicochemical effects, such as lubrication, precipitation, solubilization, ion exchange and redox^97^. Redox reactions mainly refer to the chemical corrosion of coal and rock by solutions such as acids and bases^77,98,99^. However, for this SHPB dynamic load experiment, the study of the influence of water and brine on the solid components should mainly focus on physical effects. Specifically, the impact on dry coal and rock is mainly Type I failure caused by tensile stress $\sigma_{1}$ at the crack tip^37^. When the coal or rock is immersed in water, in addition to eroding the solid medium and generating surface film tension to reduce the strength of the specimen, the free water in the crack increases the meniscus effect stress $\sigma_{2}$, Stephen effect stress $\sigma_{3}$ and Newton internal friction effect $\sigma_{4}$ on the basis of the tip tensile stress $\sigma_{1}$, thus generating resistance to crack growth under dynamic loading conditions^92^.

**Figure 15:**
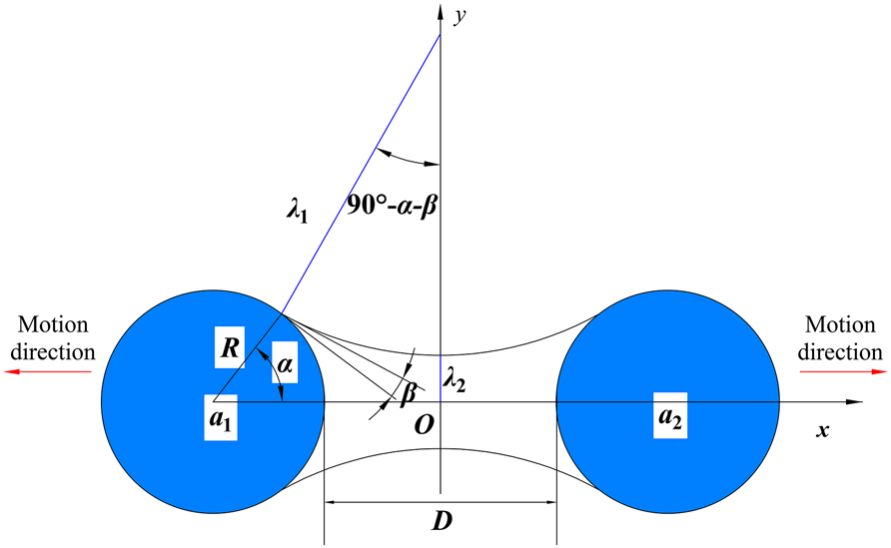
Interaction model of a solid particle and a water molecule.

To reveal the influence of free water on coal and rock fracture expansion, adjacent solid particles are simplified into spheres, and the presence of water plays an important role in the cohesion between spheres, as shown in Fig. 15. At this time, water exists between solid particles in the form of oscillating liquid bridges^100^, cohesion can be summarized as an attractive force and a liquid bridge force^101,102^, and the liquid bridge force can be divided into a static liquid bridge force and a dynamic liquid bridge force^38^, which can be expressed as:

16 $$ F_{total} = F_{\alpha } + F_{cap} + F_{vis} $$

In Eq. (16), $F_{total}$ is the dynamic total cohesion force, $F_{\alpha }$ is the attractive force between particles, and $F_{cap}$ is the static liquid bridge force (also known as the capillary force), which is mainly caused by the capillary pressure difference inside the liquid and the surface tension of the fluid, and its value is equal to the sum of the axial components of the two. $F_{vis}$ is the dynamic liquid bridge force (also known as the dynamic viscosity force), which is generated by the friction of the relative motion of particles. When the liquid bridge gravity is not considered, the expressions for $F_{\alpha }$, $F_{cap}$, and $F_{vis}$ can be further written as^41,103,104^:

17 $$ F_{\alpha } = G\frac{{m_{1} m_{2} }}{{(2R + D)^{2} }} $$

18 $$ F_{cap} = 2\pi \lambda_{2} \sigma_{s} - \pi \lambda_{{_{2} }}^{2} \sigma_{s} \left[ {\frac{1}{{\lambda_{1} }} + \frac{1}{{\lambda_{2} }}} \right] $$

19 $$ F_{vis} = \frac{3}{2}\pi \eta R^{2} \left[ {1 - \frac{D/2}{{H(b)}}} \right]^{2} \frac{1}{D/2}\frac{dD}{{dt}} $$

In Eqs. (17)–(19), $G$ is the gravitational constant; $m_{1}$ and $m_{2}$ are the masses of the two particles; $R$ is the radius of the sphere; $D$ is the distance between the surfaces of the two spheres; $\sigma_{s}$ is the solid‒liquid interfacial tension; $\beta$ is the solid‒liquid contact angle; $\eta$ is the fluid viscosity coefficient; $\lambda_{1}$ and $\lambda_{2}$ are the radii of curvature of the liquid bridge arc and liquid bridge neck, respectively; and $dD/dt$ is the particle motion velocity. According to the geometric relationship shown in Fig. 15, the expressions for the two radii of curvature are:

20 $$ \lambda_{1} = \frac{D/2 + R(1 - \cos \alpha )}{{\cos (\alpha + \beta )}} $$

21 $$ \lambda_{2} = R\sin \alpha + \frac{[\sin (\alpha + \beta ) - 1][D/2 + R(1 - \cos \alpha )]}{{\cos (\alpha + \beta )}} $$

In Eqs. (20)–(21), $\alpha$ is the angle between the liquid and spherical wetting three-phase line on the sphere, i.e., the half-filling angle.

22 $$ H(b) = D/2 + \frac{{b^{2} }}{R} $$

In Eq. (22), $b$ is the wet circumference of the particle.

Summarizing Eqs. (17)–(19) and substituting them into Eq. (16) and combining with Eqs. (20)–(21), we obtain:

23 $$ F_{total} = G\frac{{m_{1} m_{2} }}{{(2R + D)^{2} }} + 2\pi \lambda_{2} \sigma_{s} - \pi \lambda_{{_{2} }}^{2} \sigma_{s} \left[ {\frac{1}{{\lambda_{1} }} + \frac{1}{{\lambda_{2} }}} \right] + \frac{3}{2}\pi \eta R^{2} \left[ {1 - \frac{D/2}{{H(b)}}} \right]^{2} \frac{1}{D/2}\frac{dD}{{dt}} $$

In addition, by integrating the surface of the liquid bridge shown in Fig. 15 along the axial direction, the liquid bridge volume can be obtained as:

24 $$ V = 2\pi \int_{0}^{{x_{r} }} {\left( {\lambda_{1} + \lambda_{2} - \sqrt {(\lambda_{1} )^{2} - X^{2} } } \right)}^{2} dX - \frac{2}{3}\pi R^{3} (1 - \cos \alpha )^{2} (2 + \cos \alpha ) $$

According to Eqs. (23) and (24), (1) when the particle size *R* of the coal and rock particles is constant, each term in the cohesion force is inversely proportional to the surface spacing *D*. In other words, as the particle surface spacing increases, the interparticle cohesion decreases rapidly. (2) Compared to the static liquid bridge force, the remaining two parameters, especially the dynamic liquid bridge force, exhibit a more obvious tendency to decrease. The dynamic liquid bridge force approaches zero when the particle spacing reaches a small distance. (3) When the surface spacing *D* is constant, both the static liquid bridge force and the dynamic liquid bridge force are proportional to the particle size *R*. The larger the particle size is, the greater the liquid bridge force that hinders crack propagation. (4) When the surface spacing *D* is small, the total liquid bridge force decreases with increasing liquid bridge volume; when the surface spacing *D* exceeds one tenth of S_max_ (S_max_ is the critical fracture distance of the liquid bridge), the total liquid bridge force increases with increasing liquid bridge volume^105^.

In this paper, the solid particle size *R* and surface spacing *D* are actually controlled by two independent variables, namely, the porosity and moisture content. However, based on the assumption that coal and rock specimens do not absorb water per se^38^, pores and fractures are important spaces for storing water. The above mechanism can effectively explain the relevant experimental results, such as the following: (1) Compared to rock specimens, coal specimens have a higher porosity. The increase in porosity not only weakens the strength of the specimen but also increases the surface spacing *D*, thereby reducing the static and dynamic liquid bridge forces, decreasing the ability of the specimen to resist damage, and increasing the opening and speed of crack propagation, resulting in a consequent increase in the values of the two fractal dimensions, namely, the box dimension during the destruction of the specimen and the mass dimension after the destruction of the specimen. (2) When the porosity remains constant, with increasing infiltration degree of the rock specimens, the liquid bridge morphology, liquid bridge volume, and infiltration angle all change. Specifically, there is an optimal value for the liquid bridge volume. Below this value, the adhesion force between particles is positively correlated with the liquid bridge volume, gradually reaching a peak; thereafter, the adhesion force between particles is negatively correlated with the liquid bridge volume, gradually decreasing^38^. From a macroscopic perspective, with increasing moisture content, the ability of the specimen to resist impact damage first increases and then decreases, which is highly consistent with the stress‒strain curve of the rock specimens shown in Fig. 8b in the previous section. Notably, due to the greater sensitivity of the liquid bridge force to surface spacing *D*, the coal specimens do not exhibit similar patterns after soaking. (3) The SEM results indicate that specimens soaked in brine will produce more pores and cracks than those soaked in distilled water, which will also decrease the physicomechanical properties of the specimen while increasing the surface spacing and critical water absorption of the specimen, thereby increasing the dissipated energy of the specimen and further reducing its ability to resist impact damage. (4) The cohesive force between particles is mainly composed of interparticle attraction, static liquid bridge force and dynamic liquid bridge force, as shown in Eqs. (16–19). An increase in the concentration of sodium chloride in the liquid will reduce the surface tension between the liquid and the solid particles, resulting in a decrease in cohesion^106^. Moreover, the NaCl solution can also increase the contact angle between the particles and the liquid^107^, resulting in decreased stability of the specimens soaked in the NaCl solution and more cracks after impact. This requires additional attention in the engineering field.

## Conclusions

In this study, using an SHPB impact loading experimental system, the dynamic mechanical response and destruction characteristics of coal and rock under the action of distilled water and saline solution were systematically analysed, and the influences of impact velocity, moisture content, and immersion liquid characteristics on the experimental results were comprehensively revealed. The microscopic characteristics of the crack evolution and failure modes under the coupled effect of saltwater and dynamics were highlighted, and the experimental mechanism was mathematically explained. The following conclusions were drawn:

1. In this experiment, the longitudinal wave speeds of the rock and coal specimens show a typical positive correlation with the water content, and the longitudinal wave velocity of the rock specimens is greater than that of the coal specimens, both in terms of the value and growth rate with increasing water content. Under saturated soaking conditions, the longitudinal wave velocity of the saturated salt-containing specimens is greater than that of the saturated water-containing specimens.
2. At similar impact velocities, with increasing moisture content, both the dynamic compressive strength and dynamic elastic modulus of the coal specimens decrease, and the peak strain increases accordingly. In contrast, the dynamic compressive strength of the rock specimens first increases and then decreases. In addition, the effect of the immersion medium on coal bodies is much greater than that on rocks, and the weakening effect of saltwater on the physiomechanical properties of coal and rock is much greater than that of distilled water.
3. As the energy of the incident wave increases, the dissipated energy per unit volume of coal and rock also increases simultaneously, and both exhibit a strong linear correlation. Moreover, for coal, with increasing incident energy, the energy conversion efficiency of the specimen gradually decreases. In contrast, the energy conversion efficiency of the rock specimen first increases and then decreases, with a peak point.
4. Under high strain rates, the specimens with moisture content have a lower surface crack extension rate in the initial stage, and this characteristic does not change until the main crack opening increases to a certain degree. The complexity of surface cracks in coal and rock specimens immersed in saltwater is greater than that in distilled water.
5. The strain rate plays a key role in the mass frequency distribution of the failure products of the specimens. With increasing impact velocity, the failure modes of the coal and rock specimens change from splitting to crushing to smashing. With increasing moisture content, the damage degree of the specimens increases, and the proportion of large pieces after crushing gradually decreases. The mass fractal dimensions basically show a trend of fully saturated water-bearing > semisaturated water-bearing > naturally water-absorbing specimens. The final failure degree of specimens immersed in saltwater is often more severe than that of specimens immersed in distilled water.
6. SEM analysis after impact failure shows that the numbers of surface micropores and cracks in the coal and rock specimens exhibit the following characteristics: drying < fully saturated water-bearing < fully saturated salt-bearing specimens. Regardless of the state, there are fewer surface micropores and cracks in the rock specimens than in the coal specimens, and the smoothness of the rock specimens is also greater than that of the coal specimens. For the rock specimens, when the particle surface spacing is small, the total liquid bridge force decreases with increasing liquid bridge volume. When the particle surface spacing exceeds one-tenth of the critical fracture distance of the liquid bridge, the total liquid bridge force increases with increasing liquid bridge volume, but the coal specimen does not exhibit a similar pattern after immersion.

## Data Availability

The datasets used and analysed during the current study are available from the corresponding author upon reasonable request.

## References

[1] Liu JH, Zhao YL, Tan T, Zhang LY, Zhu ST, Xu F (2022). Evolution and modeling of mine water inflow and hazard characteristics in southern coalfields of China: A case of Meitanba mine. Int. J. Min. Sci. Technol..

[2] Poulsen BA, Shen B, Williams DJ, Huddlestone-Holmes C, Erarslan N, Qin J (2014). Strength reduction on saturation of coal and coal measures rocks with implications for coal pillar strength. Int. J. Rock Mech. Min. Sci..

[3] Yasidu UM, Fujii Y, Kodama JI, Fukuda D, Maneya GJ, Dandadzi J, Dassanayake AB (2019). Influences of water vapor on roof fall accidents in selected underground coal mines in Malawi. Adv. Civ. Eng..

[4] Sun WJ, Zhou WF, Jiao J (2016). Hydrogeological classification and water inrush accidents in China’s coal mines. Mine Water Environ..

[5] Cheng WM, Nie W, Zhou G, Yu YB, Ma Y, Xue J (2012). Research and practice on fluctuation water injection technology at low permeability coal seam. Saf Sci.

[6] Yan JJ, Wang F, Li YC, Gao YB, Li ZG, Liu HW (2020). A feasibility study of coal seam water injection processes: The effects of coal porosity and mass flow rates of injected water on wetting radii. Energy Fuel.

[7] Farmer IW, Attewell PB (1965). Rock penetration by high velocity water jet: A review of the general problem and an experimental study. Int. J. Rock Mech. Min. Sci. Geomech. Abstr..

[8] Wang H, Wang EY, Li ZH, Shen RX, Liu XF, Gao XY, Li B, Zhang QM (2020). Study on safety pressure of water jet breaking coal based on the characteristic analysis of electromagnetic radiation signal. Process Saf. Environ..

[9] Shi H, Zhang YB, Tang L (2021). Physical test of fracture development in the overburden strata above the goaf and diffusion process of permeable grout slurry. Bull Eng. Geol. Environ..

[10] Ao XF, Wang XL, Zhu XB, Zhou ZY, Zhang XX (2017). Grouting simulation and stability analysis of coal mine goaf considering hydromechanical coupling. J. Comput. Civil. Eng..

[11] Fan J, Dou LM, He H, Du TT, Zhang SB, Gui B, Sun XL (2012). Directional hydraulic fracturing to control hard-roof rockburst in coal mines. Int. J. Min. Sci. Technol..

[12] Liu JW, Liu CY, Yao QL, Si GY (2020). The position of hydraulic fracturing to initiate vertical fractures in hard hanging roof for stress relief. Int. J. Rock. Mech. Min..

[13] Lan TW, Fan CJ, Zhang HW, Batugin AS, Ruibin L, Yang Y, Jia C (2018). Seepage law of injected water in the coal seam to prevent rock burst based on coal and rock system energy. Adv. Civ. Eng..

[14] Lin BQ, Shen CM (2015). Coal permeability-improving mechanism of multilevel slotting by water jet and application in coal mine gas extraction. Environ. Earth Sci..

[15] Liu XF, Xu G, Zhang C, Kong B, Qian JF, Zhu D, Wei MY (2017). Time effect of water injection on the mechanical properties of coal and its application in rockburst prevention in mining. Energies.

[16] Xie J, Liang YP, Zou QL, Li L, Li XL (2019). Elimination of coal and gas outburst risk of low-permeability coal seam using high-pressure water jet slotting technology: A case study in Shihuatian Coal Mine in Guizhou Province, China. Energy Sci. Eng..

[17] Ray SK, Singh RP (2007). Recent developments and practices to control fire in undergound coal mines. Fire Technol..

[18] Zhou G, Xu M, Fan T (2018). Numerical simulation of combined water injection in deep coal seam and its field application for dust suppression and de-stressing: A case study at dongtan coal mine, China. Geotech. Geol. Eng..

[19] Gu DZ (2015). Theory framework and technological system of coal mine underground reservoir. J. Chin. Coal. Soc..

[20] Zhang C, Wang FT, Bai QS (2021). Underground space utilization of coalmines in China: A review of underground water reservoir construction. Tunn. Undergr. Sp. Tech..

[21] Fan JY, Xie HP, Chen J, Jiang DY, Li CB, Tiedeu WN, Ambre J (2020). Preliminary feasibility analysis of a hybrid pumped-hydro energy storage system using abandoned coal mine goafs. Appl. Energy.

[22] Li HR, Qiao YF, He MC, Shen RX, Gu ZJ, Cheng T, Xiao YM, Tang J (2023). Effect of water saturation on dynamic behavior of sandstone after wetting-drying cycles. Eng. Geol..

[23] Zhao YC, Yang TH, Xu T, Zhang PT, Shi WH (2018). Mechanical and energy release characteristics of different water-bearing sandstones under uniaxial compression. Int. J. Damage Mech..

[24] Ju F, Wang D, Wang ZW, Xiao M, He ZQ, Ning P, Wang TF, Zhou C, Zhang YZ, Yan CS (2022). Research on mechanical properties and damage constitutive model of water-bearing coal. Appl. Sci..

[25] Luo Y, Gong FQ, Zhu CQ (2022). Experimental investigation on stress-induced failure in D-shaped hard rock tunnel under water-bearing and true triaxial compression conditions. Bull. Eng. Geol. Environ..

[26] Zhang MC, Nie RS (2020). Experimental investigation on the influence of water content on the mechanical properties of coal under conventional triaxial compression. Shock Vib..

[27] Zhou ZL, Cai X, Cao WZ, Li XB, Xiong C (2016). Influence of water content on mechanical properties of rock in both saturation and drying processes. Rock Mech. Rock Eng..

[28] Tang CJ, Yao QL, Li ZY, Zhang Y, Ju MH (2019). Experimental study of shear failure and crack propagation in water-bearing coal samples. Energy Sci. Eng..

[29] Zhu J, Deng JH (2023). Insights from a combined analysis of acoustic emission signals for water-bearing rocks in four-point bending tests: Failure mode classification and strength degradation. Rock Mech. Rock Eng..

[30] Zhou ZL, Cai X, Ma D, Cao WZ, Chen L, Zhou J (2018). Effects of water content on fracture and mechanical behavior of sandstone with a low clay mineral content. Eng. Fract. Mech..

[31] Zhou ZL, Cai X, Ma D, Du X, Chen L, Wang H, Zang HZ (2019). Water saturation effects on dynamic fracture behavior of sandstone. Int. J. Rock Mech. Min..

[32] van Eeckhout EM (1976). The mechanisms of strength reduction due to moisture in coal mine shales. Int. J. Rock Mech. Min. Sci. Geomech..

[33] Cai X, Zhou ZL, Liu KW, Du XM, Zang HZ (2019). Water-weakening effects on the mechanical behavior of different rock types: Phenomena and mechanisms. Appl. Sci..

[34] Sun XY, Jin TX, Li JH, Xie JL, Li CT, Li XX (2023). Dynamic characteristics and crack evolution laws of coal and rock under split Hopkinson pressure bar impact loading. Meas. Sci. Technol..

[35] Li XB, Lok TS, Zhao J (2005). Dynamic characteristics of granite subjected to intermediate loading rate. Rock Mech. Rock Eng..

[36] Man K, Liu XL, Song ZF, Liu ZX, Liu RL, Wu LW, Cao ZX (2022). Dynamic compression characteristics and failure mechanism of water-saturated granite. Water.

[37] Gu HL, Tao M, Li XB, Li QY, Cao WZ, Wang F (2018). Dynamic response and failure mechanism of fractured coal under different soaking times. Theor. Appl. Fract. Mech..

[38] Gu HL, Tao M, Cao WZ, Zhou J, Li XB (2019). Dynamic fracture behaviour and evolution mechanism of soft coal with different porosities and water contents. Theor. Appl. Fract. Mech..

[39] Gu HL, Tao MM, Li XM, Cao WZ, Li QY (2020). Dynamic tests and mechanical model for water-saturated soft coal with various particle gradations. Int. J. Rock Mech. Min..

[40] Zhang XH, Chiu YW, Hao H, Cui J (2023). Dynamic enhancing effect of free water on the dynamic tensile properties of mortar. Mater. Struct..

[41] Wang K, Feng GR, Bai JW, Guo J, Shi XD, Cui BQ, Song C (2022). Dynamic behaviour and failure mechanism of coal subjected to coupled water-static-dynamic loads. Soil Dyn. Earthq. Eng..

[42] Shan CH, Yao QL, Cao SG, Xie HX, Xu Q, Zheng CK, Chen XY (2023). Measurement of fracture development evolution of coal samples under acid-alkaline by three-dimensional reconstruction and AE time-frequency characteristic analysis. Measurement.

[43] Si LL, Xun YJ, Wei JP, Li B, Wang HY, Yao BH, Liu Y (2022). Dissolution characteristics of gas in mine water and its application on gas pressure measurement of water-intrusion coal seam. Fuel.

[44] Dong FY, Yin HY, Cheng WJ, Li YJ, Qiu M, Zhang CW, Tang RQ, Xu GL, Zhang LF (2022). Study on water inrush pattern of Ordovician limestone in North China Coalfield based on hydrochemical characteristics and evolution processes: A case study in Binhu and Wangchao Coal Mine of Shandong Province, China. J. Clean Prod..

[45] Xu JY, Gui HR, Chen JY, Li C, Li Y, Zhao C, Guo Y (2022). Hydrogeochemical characteristics and formation mechanisms of the geothermal water in the Qingdong coal mine, Northern Anhui Province, China. Mine Water Environ..

[46] Xu XM, Liu JF, Jin XF, Zhang YH, Arif M, Wang C, Iglauer S (2022). Dynamic mechanical response characteristics of coal upon exposure to KCl brine. Geomech. Geophys. Geo..

[47] Xu D, Gao MS, Zhao YC, He YL, Yu X (2020). Study on the mechanical properties of coal weakenedby acidic and alkaline solutions. Adv Civ Eng.

[48] Li T, Tang YS, Li LH, Hu HY, Li Z, He JQ, An BC (2023). Study of the catastrophic process of water–sand inrush in a deep buried stope with thin bedrock. Water.

[49] Wang ZH, Wu SX, Li JL, Sun WC, Wang ZF, Liu PJ (2023). Surface subsidence and its reclamation of a coal mine locating at the high groundwater table, China. Int. J. Environ. Sci. Technol..

[50] Miao W, Xu YC, Guo Y, Zhang EM, Zhuo YL, Huang L, Ma ZM, Liang SY (2022). The hydrogeological characteristics of thick alluvium with high water level and the influence on Zhaogu mining area, Henan Province, China. Geofluids.

[51] Zhang S, Tang SJ, Zhang DS, Fan GW, Wang Z (2017). Determination of the height of the water-conducting fractured zone in difficult geological structures: A case study in Zhao Gu No. 1 coal seam. Sustainability.

[52] Xu YC, Luo YQ, Li JH, Li KQ, Cao XC (2018). Water and sand inrush during mining under thick unconsolidated layers and thin bedrock in the Zhaogu No 1 Coal Mine, China. Mine Water Environ..

[53] Wang JC, Wang ZH, Tang YS, Li M, Chang KL, Gong H, Xu GL (2021). Experimental study on mining-induced dynamic impact effect of main roofs in deeply buried thick coal seams with weakly consolidated thin bed rock. Chin. J. Rock Mech. Eng.

[54] Zhou YX, Xia KW, Li XB, Li HB, Ma GW, Zhao J, Zhou ZL, Dai F (2011). Suggested methods for determining the dynamic strength parameters and mode-I fracture toughness of rock materials. Int. J. Rock Mech. Min..

[55] Sun XY, Chen GY, Li JH, Xu XM, Fu S, Xie JL, Liang L (2020). Propagation characteristics of ultrasonic P-wave velocity in artificial jointed coal briquettes. J. Geophys. Eng..

[56] ASTM 2016 E11–16 (2016). Standard Specification for Woven Wire Test Sieve Cloth and Test Sieves.

[57] Cai X, Zhou ZL, Zang HZ, Song ZY (2020). Water saturation effects on dynamic behavior and microstructure damage of sandstone: Phenomena and mechanisms. Eng. Geol..

[58] Jiang BB, Zhao Z, Liu DQ, Cao ZQ, Tang JW, Wu M, Liang DC (2023). Study on the interaction mechanism between residual coal and mine water in goaf of coal mine underground reservoir. Sustainability.

[59] Rawat NS (1976). Corrosivity of underground mine atmospheres and mine waters: A review and preliminary study. Br. Corros. J..

[60] Xu D, Gao MS, Zhao YC, He YL, Yu X (2020). Study on the mechanical properties of coal weakenedby acidic and alkaline solutions. Adv. Civ. Eng..

[61] Tang CJ, Yao QL, Chen T, Shan CH, Li J (2022). Effects of water content on mechanical failure behaviors of coal samples. Geomech. Geophys. Geo-Energy Geo-Resour..

[62] Zhang ZX, Kou SQ, Jiang LG, Lindqvist PA (2000). Effects of loading rate on rock fracture: Fracture characteristics and energy partitioning. Int. J. Rock Mech. Min..

[63] Yan ZL, Dai F, Liu Y, Du HB (2020). Experimental investigations of the dynamic mechanical properties and fracturing behavior of cracked rocks under dynamic loading. Bull. Eng. Geol. Environ..

[64] Weng L, Wu ZJ, Liu QS, Wang ZY (2019). Energy dissipation and dynamic fragmentation of dry and water-saturated siltstones under sub-zero temperatures. Eng. Fract. Mech..

[65] Ding CX, Yang RS, Yang LY (2021). Experimental results of blast-induced cracking fractal characteristics and propagation behavior in deep rock mass. Int. J. Rock Mech. Min..

[66] Zhai C, Xu JZ, Liu SM, Qin L (2018). Fracturing mechanism of coal-like rock specimens under the effect of non-explosive expansion. Int. J. Rock Mech. Min..

[67] Wang LC, Duan K, Zhang QY, Li XJ, Jiang RH (2022). Study of the dynamic fracturing process and stress shadowing effect in granite sample with two holes based on SCDA fracturing method. Rock Mech. Rock Eng..

[68] Ai T, Zhang R, Zhou HW, Pei JL (2014). Box-counting methods to directly estimate the fractal dimension of a rock surface. Appl. Surf. Sci..

[69] Sun B, Liu S, Zeng S, Wang SY, Wang SP (2021). Dynamic characteristics and fractal representations of crack propagation of rock with different fissures under multiple impact loadings. Sci. Rep.-UK.

[70] Zhao YH, Huang JF, Wang R (1993). Fractal characteristics of meso-fractures in compressed rock specimens. Int. J. Rock Mech. Min. Sci. Geomech. Abstr..

[71] Ai DH, Zhao YC, Wang QF, Li CW (2019). Experimental and numerical investigation of crack propagation and dynamic properties of rock in SHPB indirect tension test. Int. J. Impact. Eng..

[72] Bagde MN, Raina AK, Chakraborty AK, Jethwa JL (2002). Rock mass characterization by fractal dimension. Eng. Geol..

[73] Yang XL, Wang L, Yu MG, Chu TX, Li HT, Chao JK, Han XF (2022). Experimental study on the secondary breakup properties of crushed coal through a dynamic porosity model based on Weibull distribution theory. Fuel.

[74] Shen X, Shen Y, Xu JH, Liu HL (2022). Influence of the fractal distribution of particle size on the critical state characteristics of calcareous sand. Fractal Fract..

[75] Peng RD, Ju Y, Wang JG, Xie HP, Gao F, Mao LT (2015). Energy dissipation and release during coal failure under conventional triaxial compression. Rock Mech. Rock Eng..

[76] Knight R, Nolen-Hoeksema R (1990). A laboratory study of the dependence of elastic wave velocities on pore scale fluid distribution. Geophys. Res. Lett..

[77] Li L, Fan JB, Liu NN, Gong S, Yang DM (2021). Effect of acid and alkaline environment on dynamic strength and porosity characteristics of bursting liability coal. Shock Vib..

[78] Bergsaker AS, Røyne A, Ougier-Simonin A, Aubry J, Renard F (2016). The effect of fluid composition, salinity, and acidity on subcritical crack growth in calcite crystals. J. Geophys. Res. Sol. Earth.

[79] Çelik MY, Kaçmaz AU (2016). The investigation of static and dynamic capillary by water absorption in porous building stones under normal and salty water conditions. Environ. Earth Sci..

[80] Best AI, McCann C, Sothcott J (1994). The relationships between the velocities, attenuations and petrophysical properties of reservoir sedimentary rocks. Geophys. Prospect..

[81] Kahraman S (2007). The correlations between the saturated and dry P-wave velocity of rocks. Ultrasonics.

[82] Zhou CY, Deng YM, Tan XS, Liu ZQ, Lin CX (2003). Research on the variation regularities of microstructures in the testing of interaction between soft rocks and water. Acta Sci. Nat. Univ. Sunyatseni.

[83] Zheng D, Li QB (2004). An explanation for rate effect of concrete strength based on fracture toughness including free water viscosity. Eng. Fract. Mech..

[84] Jin TX, Sun XY, Liu K, Lin SR, Yang SQ, Xie JL (2023). Experimental study of the multiple fractalisation of coal and rock failure subjected to the coupled effects of water, temperature and dynamic loads. Appl. Sci..

[85] Zhao YH (1998). Crack pattern evolution and a fractal damage constitutive model for rock. Int. J. Rock Mech. Min..

[86] Liu ZX, Han KW, Yang S, Liu YX (2020). Fractal evolution mechanism of rock fracture in undersea metal mining. J. Cent. South Univ..

[87] Gao Y, Yu ZX, Chen WQ, Yin Q, Wu JY, Wang W (2023). Recognition of rock materials after high-temperature deterioration based on SEM images via deep learning. J. Mater. Res. Technol..

[88] Iakovidis DK, Goudas T, Smailis C, Maglogiannis I (2014). Ratsnake: A versatile image annotation tool with application to computer-aided diagnosis. Sci. World J..

[89] Ai DH, Zhao YC, Wang QF, Li CW (2020). Crack propagation and dynamic properties of coal under SHPB impact loading: Experimental investigation and numerical simulation. Theor. Appl. Fract. Mech..

[90] Makarov PV, Smolin I, Cherepanov OI, Trubitsyna NV, Voroshilov YS (2002). Elastic viscoplastic deformation and fracture of coal at the mesoscopic scale. Phys. Mesomech..

[91] Zhao YX, Zhao GF, Jiang YD (2013). Experimental and numerical modelling investigation on fracturing in coal under impact loads. Int. J. Fract..

[92] Chen Y, Kang T, Wu C (2023). Study on mechanical behavior and mechanism of sandstone under the coupling effect of water content and dynamic load. Processes.

[93] Zheng, D. Study on rock micro-rupture mechanism and macroscopic fracture characteristic of phyllite, in *International Conference on Consumer Electronics, Communications and Networks (CECNet)*, 1715–1718 (Curran Associates, 2011).

[94] Wang YB (2018). Rock dynamic fracture characteristics based on NSCB impact method. Shock Vib..

[95] Zhang YH, Lebedev M, Al-Yaseri A, Yu HY, Xu XM, Sarmadivaleh M, Barifcani A, Iglauer S (2018). Nanoscale rock mechanical property changes in heterogeneous coal after water adsorption. Fuel.

[96] Yao YB, Liu DM, Che Y, Tang DZ, Tang SH, Huang WH (2009). Non-destructive characterization of coal samples from China using microfocus X-ray computed tomography. Int. J. Coal Geol..

[97] Zhang C, Bai QS, Han PH, Wang L, Wang XJ, Wang FT (2023). Strength weakening and its micromechanism in water–rock interaction, a short review in laboratory tests. Int. J. Coal Sci. Technol..

[98] Hutchinson AJ, Johnson JB, Thompson GE, Wood GC, Sage PW, Cooke MJ (1993). Stone degradation due to wet deposition of pollutants. Corros. Sci..

[99] Feng XT, Chen SL, Zhou H (2004). Real-time computerized tomography (CT) experiments on sandstone damage evolution during triaxial compression with chemical corrosion. Int. J. Rock Mech. Min..

[100] Fisher RA (1926). On the capillary forces in an ideal soil; correction of formulae given by WB Haines. J. Agr. Sci..

[101] Soulie F, Cherblanc F, El Youssoufi MS, Saix C (2006). Influence of liquid bridges on the mechanical behaviour of polydisperse granular materials. Int. J. Numer. Anal. Met..

[102] Tsunazawa Y, Fujihashi D, Fukui S, Sakai M, Tokoro C (2016). Contact force model including the liquid-bridge force for wet-particle simulation using the discrete element method. Adv. Powder Technol..

[103] Adams MJ, Perchard V (1985). The cohesive forces between particles with interstitial liquid. Inst. Chem. Eng. Symp..

[104] Chan DY, Horn RG (1985). The drainage of thin liquid films between solid surfaces. J. Chem. Phys.

[105] Lian G, Thornton C, Adams MJ (1993). A theoretical study of the liquid bridge forces between two rigid spherical bodies. J. Colloid Interface Sci..

[106] Wang M, Tian YK, Zhang ZJ, Guo QF, Wu LL (2023). Dynamic evolution of coal pore-fracture structure and its fractal characteristics under the action of salty solution. Mathematics.

[107] Sghaier N, Prat M, Nasrallah SB (2006). On the influence of sodium chloride concentration on equilibrium contact angle. Chem. Eng. J..

